# Harnessing Beneficial Microbes and Sensor Technologies for Sustainable Smart Agriculture

**DOI:** 10.3390/s25216631

**Published:** 2025-10-29

**Authors:** Younes Rezaee Danesh

**Affiliations:** Department of Plant Protection, Faculty of Agriculture, Van Yuzuncu Yil University, Van 65090, Türkiye; y.rdanesh@yahoo.com

**Keywords:** beneficial microbes, sensor technologies, sustainable agriculture, precision farming, soil health

## Abstract

The integration of beneficial microorganisms with sensor technologies represents a transformative advancement toward sustainable smart agriculture. This review synthesizes recent progress in combining microbial bioinoculants with sensor-based monitoring systems to enhance crop productivity, resource-use efficiency, and environmental resilience. Beneficial bacteria and fungi improve nutrient cycling, stress tolerance, and soil fertility thereby reducing the reliance on chemical fertilizers and pesticides. In parallel, sensor networks—including soil moisture, nutrient, environmental, and remote-sensing platforms—enable real-time, data-driven management of agroecosystems. Integrated microbe–sensor approaches have demonstrated 10–25% yield increases and up to 30% reductions in agrochemical inputs under optimized field conditions. We propose an integrative Microbe–Sensor Closed Loop (MSCL) framework in which microbial activity and sensor feedback interact dynamically to optimize inputs, monitor plant–soil interactions, and sustain productivity. Key applications include precision fertilization, stress diagnostics, and early detection of nutrient or pathogen imbalances. The review also highlights barriers to large-scale adoption, such as variable field performance of inoculants, high sensor costs, and limited interoperability of data systems. Addressing these challenges through standardization, cross-disciplinary collaboration, and farmer training will accelerate the transition toward climate-smart, self-regulating agricultural systems. Collectively, the integration of biological and technological innovations provides a clear pathway toward resilient, resource-efficient, and ecologically sound food production.

## 1. Introduction to Smart Agriculture

Smart agriculture represents a transformative paradigm that integrates beneficial microbes, sensor technologies, and data-driven management systems to optimize agricultural productivity while minimizing environmental impact. Unlike conventional farming, which depends heavily on chemical fertilizers and pesticides, smart agriculture promotes resource efficiency, cost-effectiveness, and environmental sustainability—priorities that are increasingly critical in the context of global climate change [1]. Traditional practices are often associated with excessive input use, high operational costs, and ecological degradation, whereas smart farming leverages Internet of Things (IoT)-based sensor networks to monitor key environmental variables such as soil moisture, temperature, humidity, and pH in real time [2]. These interconnected systems transmit data to user-friendly digital platforms, enabling farmers to determine optimal irrigation and fertilization schedules with unprecedented precision. When coupled with plant growth-promoting microorganisms (PGPMs)—including rhizobacteria, mycorrhizal fungi, and other beneficial soil microbes—sensor-guided management creates a synergistic framework that enhances nutrient uptake, stress tolerance, and soil fertility, while simultaneously reducing dependence on synthetic agrochemicals [3]. This biologically informed precision farming not only improves crop yield and quality but also minimizes harmful residues in soil and water, thereby advancing food safety and ecological resilience. The successful realization of smart agriculture depends on interdisciplinary collaboration among experts in biology, agronomy, engineering, information technology, and policy development. Such partnerships are essential for translating scientific innovations, scalable applications ensuring both technology transfer and farmer empowerment. Ultimately, the integration of microbial biotechnologies with intelligent sensor systems offers a holistic pathway toward sustainable food production, enabling agriculture to adapt to environmental stressors, conserve natural resources, and secure long-term global food security [4].

## 2. The Role of Beneficial Microbes in Agriculture

**Figure 1:**
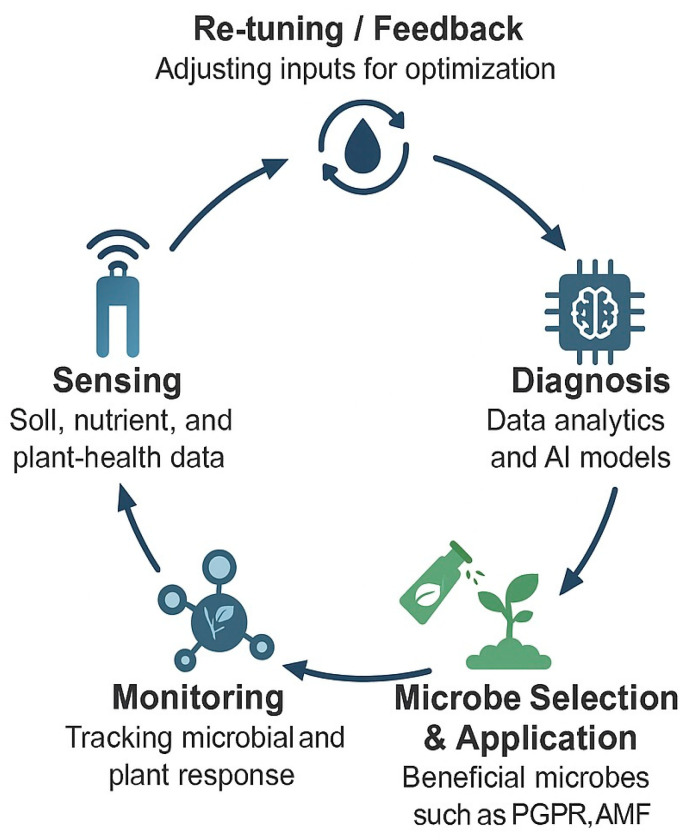
Conceptual framework of the Microbe–Sensor Closed Loop (MSCL), illustrating the continuous feedback cycle between sensor-based diagnosis and microbial application for adaptive, data-driven management in sustainable smart agriculture. The framework integrates real-time sensor data (e.g., soil moisture, nutrient levels, and plant physiological parameters) with microbial interventions such as biofertilizers, biostimulants, and biocontrol agents. Arrows represent the bidirectional information and response flow between sensing systems and microbial applications, forming a closed feedback loop that enables precision adjustment of inputs based on field data. This conceptual model underlines how linking biological processes with sensor networks can enhance resource efficiency, productivity, and environmental sustainability.

Beneficial microbes—including diverse groups of bacteria, fungi, and actinomycetes—are fundamental to sustainable agriculture due to their multifunctional roles in enhancing crop productivity and environmental resilience. These microorganisms improve plant performance through several mechanisms, such as pathogen suppression, nutrient solubilization, nitrogen fixation, phytohormone synthesis, and stress alleviation [5,6]. Among them, plant growth-promoting rhizobacteria (PGPR) and arbuscular mycorrhizal fungi (AMF) are particularly significant. PGPR colonize the rhizosphere and stimulate plant growth through direct mechanisms (e.g., auxin and gibberellin production) and indirect mechanisms, including antibiosis and induced systemic resistance. Meanwhile, AMF expand the effective root surface area, facilitating the uptake of immobile nutrients such as phosphorus and zinc, and improving water-use efficiency, particularly under drought or salinity stress [7,8,9]. Beyond their role in plant physiology, beneficial microbes contribute to soil restoration by enhancing organic matter decomposition and accelerating nutrient cycling. Their ecological functions reduce dependence on chemical fertilizers and pesticides, thereby mitigating environmental pollution and preserving soil biodiversity. For instance, microbial inoculation with *Rhizobium*, *Azospirillum*, or *Trichoderma* species has been shown to increase crop yield by 15–35% under field conditions. Similarly, AMF and phosphate-solubilizing bacteria (PSB) can enhance nitrogen and phosphorus uptake efficiency by 20–40%, leading to a significant reduction in synthetic fertilizer use [6,10,11]. For more than a century, microbial inoculants—including biofertilizers, biostimulants, and biopesticides—have been integral to sustainable crop management worldwide. Recent advances in molecular biology continue to expand their applicability and performance [10,11]. The integration of these biological solutions with sensor-based technologies provides new opportunities for precision and adaptability in modern farming systems. Sensors that monitor soil nutrients, moisture, and temperature can deliver real-time data to determine the optimal timing and conditions for microbial inoculants application. Likewise, biosensors tracking microbial activity, soil respiration or enzyme kinetics enable farmers to assess inoculant efficiency and soil vitality directly in the field. Thus, combining microbial inoculants with continuous sensor feedback establishes the foundation of microbe–sensor-assisted agriculture—a dynamic, data-driven approach that enhances productivity while maintaining ecological integrity (Figure 1).

### 2.1. Types of Beneficial Microbes

Beneficial microbes can be broadly categorized into four main functional groups— growth promoters, biofertilizers, biopesticides, and bioremediators—each playing a distinctive complementary role in sustainable crop production [12]. Growth-promoting microorganisms enhance plant vigor through direct mechanisms, such as the biosynthesis of phytostimulators, phytohormones (e.g., auxins, gibberellins, and cytokinins), and siderophores that facilitate iron acquisition. These compounds stimulate root development, increase biomass accumulation, and improve nutrient assimilation, collectively boosting crop productivity [13]. Biofertilizers play a central role in nutrient cycling, particularly through nitrogen fixation, phosphorus solubilization, and potassium mobilization, which together contribute to the development of efficient biofertilizer formulations for various crops [14]. Microbial inoculants such as *Rhizobium*, *Azotobacter*, and phosphate-solubilizing bacteria (PSB) enrich soil fertility and promote efficient resource utilization. With the support of nutrient and moisture sensors, the timing and dosage of biofertilizer application can be optimized based on real-time soil data, improving both microbial viability and crop response. Biopesticides exert their beneficial effects indirectly by inhibiting or suppressing plant pathogens through antibiosis, competition, enzyme production, or induced systemic resistance. Prominent genera such as *Trichoderma*, *Bacillus*, and *Pseudomonas* produce antibiotics, hydrolytic enzymes, antifungal metabolites, volatile organic compounds (VOCs), and other bioactive molecules that limit pathogen activity. The integration of biosensors for rapid pathogen detection allows targeted and timely application of these microbial agents, thereby reducing unnecessary pesticide use and minimizing environmental contamination. Bioremediating microbes maintain soil and crop quality by degrading or transforming persistent pollutants such as hydrocarbons, pesticides, and heavy metals. These microorganisms convert toxic residues into harmless metabolites, restoring soil function and ecosystem balance [11]. Advanced sensor technologies, particularly those monitoring soil respiration, gas exchange, or CO_2_ flux, can track the efficiency of bioremediation processes in situ, ensuring precise and sustainable management. Together, these microbial groups form the biological backbone of smart agriculture, where sensor-assisted monitoring and precision management significantly enhance microbial performance, soil health, and long-term agricultural sustainability. The following section (Section 2.2) explores the key biological and biochemical mechanisms through which these microbes interact with plants and the environment, forming the foundation of integrated smart-farming frameworks.

### 2.2. Mechanisms of Action

Smallholder agriculture, a cornerstone of global food security, comprises diverse cropping systems that rely on resource-efficient and locally adapted management practices. Understanding the mechanisms of action (MoA) of beneficial microbes is essential for designing technology-guided, field-applicable interventions suited to these systems. Four principal and often overlapping mechanisms—resource acquisition, hormonal modulation, pathogen antagonism, and abiotic-stress tolerance—collectively determine the agronomic effectiveness of microbial inoculants [15]. Integrating these biological processes with sensor-based monitoring enables real-time assessment of microbial performance and crop responses, advancing data-driven sustainable agriculture.

#### 2.2.1. Enhanced Resource Acquisition

Beneficial microbes improve the availability and uptake of essential nutrients by developing fine filamentous networks that penetrate soil pores and mobilize ions through the secretion of organic acids, enzymes, and chelating compounds [16]. Their small cell size and high surface-area-to-volume ratio facilitate rapid nutrient scavenging, while spore-forming species survive under adverse environmental conditions. Specialized taxa target macronutrients such as phosphorus, nitrogen, and potassium, forming the biochemical foundation of biofertilizer efficiency. Modern soil and nutrient sensors can now quantify these nutrient dynamics by tracking fluctuations in soil conductivity and ion concentrations, thereby identifying the optimal timing for microbial application and fertilization.

#### 2.2.2. Hormonal Regulation and Signaling

Microbes synthesize phytohormones and related signaling molecules—such as auxins, cytokinins, gibberellins, and homoserine lactones—that regulate root architecture, flowering, and stress adaptation [17]. Early stimulation of root initiation enhances water and nutrient uptake capacity, improving plant growth and resilience. Sensor networks that measure root-zone moisture, growth rate, or nutrient content can be integrated with these biological indicators to validate, in real time, the physiological impacts of hormone-modulating microbial strains.

#### 2.2.3. Pathogen Antagonism and Biocontrol

Biocontrol microbes suppress pathogens through parasitism, competition, antibiosis, and the secretion of hydrolytic enzymes or volatile organic compounds (VOCs). Genera such as *Trichoderma*, *Bacillus*, and *Pseudomonas* demonstrate broad-spectrum antagonistic activity against root and foliar diseases, reducing dependence on synthetic pesticides. Advanced biosensors and optical imaging systems capable of detecting pathogen-specific metabolites or stress biomarkers enable precision deployment of biocontrol agents exactly when and where infection risk is highest. Nevertheless, careful strain selection and compatibility testing are essential, as antagonistic interactions among mixed inoculants can undermine efficacy and complicate field management.

#### 2.2.4. Tolerance to Abiotic Stress

Beneficial microbes enhance crop resilience to abiotic stresses such as drought, salinity, pH variation, and heavy-metal toxicity by modulating osmolyte balance, producing stress-protective metabolites, and improving soil aggregation and structure [10,11,18]. Commercial inoculants are increasingly formulated for stress-prone environments, including unprotected greenhouse systems, where stress intensity can be severe. Sensors technologies monitoring soil moisture, temperature, and ionic concentration provide essential feedback for adjusting irrigation schedules, microbial formulation dosages, or nutrient inputs to maintain microbial viability and plant performance.

Collectively, these mechanisms act synergistically to strengthen plant growth, enhance productivity, and reduce dependence on agrochemicals. When guided by sensor-based feedback, they form a biologically informed, data-driven framework capable of significantly improving the sustainability, efficiency, and profitability of modern agriculture. The main microbial groups and their mechanisms of action contributing to sustainable agriculture are summarized in Table 1.

### 2.3. Applications in Crop Production

Agricultural productivity depends on the balanced interaction of fundamental resources such as light, water, and fertile soil. However, industrialization and intensive agricultural practices have substantially increased the use of chemical fertilizers and pesticides, resulting in negative impacts on soil, water, and air quality. These challenges underscore the urgent need to develop sustainable and smart agricultural systems that align with the environmental objectives of the UN 2030 Agenda and the principles of climate-smart farming. Achieving these goals requires technologies adapted to local agroecological conditions and the active participation of farmers, communities, and entrepreneurs in technology adoption [19]. Within this context, beneficial microbes provide a powerful and eco-friendly tool to enhance plant growth, improve nutrient use efficiency, and suppress pests and phytopathogens. Their use effectively reduces dependence on synthetic agrochemicals while maintaining soil fertility and long-term productivity. Commercial formulations containing beneficial strains such as *Bacillus* spp., *Pseudomonas* spp., and *Trichoderma* spp. are already widely utilized, demonstrating consistent performance and broad adaptability across diverse cropping systems worldwide [20]. At the same time, sensor-based technologies are revolutionizing precision agriculture by providing real-time data on soil moisture, nutrient dynamics, and pest activity. Advanced nanobiosensors and nanoformulations enable targeted delivery of microbial inoculants and agrochemicals, thereby enhancing efficiency and minimizing environmental contamination [21]. Many of these sensors are geo-referenced and integrated into decision-support systems that process environmental parameters—including temperature, humidity, pH, and electrical conductivity—to guide on-site management strategies. The combination of microbial inoculants and sensor platforms gives rise to Smart-MIP (Microbial- and Sensor-Based Integrated Precision) systems, which link microbial performance data with sensor feedback to create adaptive management cycles. These systems improve fertilizer and irrigation efficiency, optimize microbial application timing, and enhance pest and disease suppression. As a result, the integrated use of microbes and sensors not only maintains or increases crop yields, but also minimizes the use of fertilizers, pesticides, and water, reinforcing agricultural sustainability, environmental safety, and climate resilience [10,22,23].

## 3. Sensor Technologies in Agriculture

Modern sensor technologies play a pivotal role in transforming traditional agriculture into a data-driven, efficient, and sustainable system. Recent advances—particularly in nano-electrochemical sensor—have led to the development of low-cost, highly sensitive, and rapid-response devices capable of real-time detection of key biological and environmental parameters [24]. These sensors can measure plant physiological signals, detect pesticide residues, and identify pathogens that threaten crops and livestock. In addition, soil nutrients such as nitrogen, phosphorus, and potassium can now be quantified within seconds, facilitating precision fertilization and soil health management. The integration of Internet of Things (IoT) connectivity allows for remote operation and continuous data transmission, enabling even farmers in remote regions to access accurate, real-time field information. Electrochemical sensor platforms, in particular, exhibit high antimicrobial sensitivity and broad adaptability across diverse agroecosystems [25]. When combined with machine learning algorithms, these sensors generate predictive insights that optimize irrigation, fertilization, and microbial inoculant application schedules. Multisensor arrays can simultaneously monitor plant and soil parameters such as nutrient content, moisture, light exposure, disease incidence, and air quality, while livestock sensors track physiological and behavioral responses. Together, these technologies enable on-site, real-time farm management and contribute significantly to resource-use efficiency and sustainability [26]. Furthermore, recent innovations in biosensors and nanobiosensors have been integrated with gene-editing tools, bioelectronics, and nanomaterials, forming the foundation of Agriculture 4.0—an advanced paradigm in which sensors interact dynamically with microbial agents to optimize field operations and productivity [23,27].

### 3.1. Types of Sensors Used

**Figure 2:**
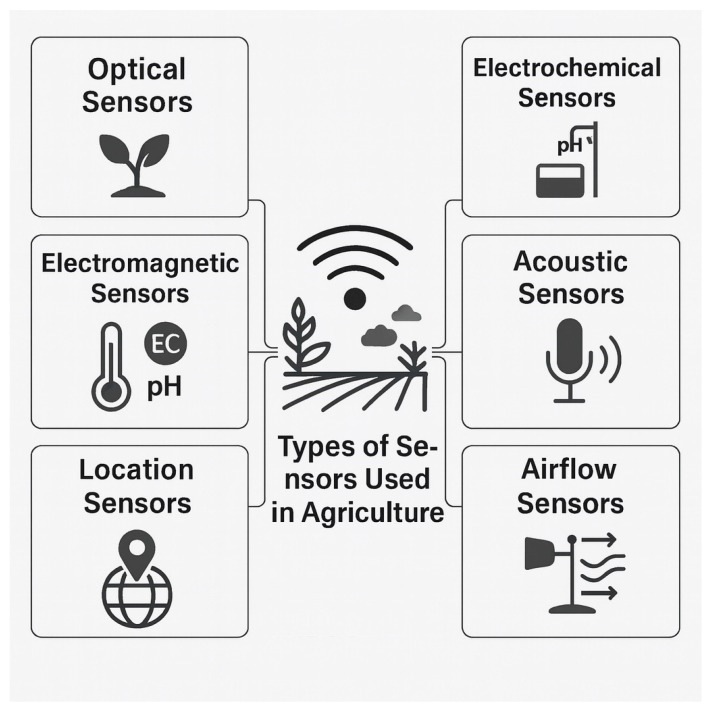
Major categories of sensors used in smart agriculture and their key monitoring parameters. The diagram classifies sensors into optical, electromagnetic, electrochemical, location-based, acoustic, and airflow types, each serving distinct diagnostic functions. Optical sensors measure reflectance indices such as NDVI and chlorophyll fluorescence to assess plant health and photosynthetic activity. Electromagnetic sensors detect soil conductivity and moisture content, providing indirect estimates of salinity and nutrient availability. Electrochemical sensors quantify ions, pH, and dissolved oxygen in soil or irrigation water, offering real-time nutrient and chemical profiling. Location sensors (GPS/GNSS) support geospatial mapping of variability across fields, while acoustic sensors are used to monitor animal welfare and pest movement. Airflow and gas sensors measure greenhouse gases (CO_2_, NH_3_, CH_4_) and microclimate parameters, contributing to environmental monitoring. Collectively, these technologies generate high-resolution, time-sensitive datasets that underpin precision, automated decision-making, and sustainable resource management in smart agriculture.

Sensor technologies form the core component of smart agriculture by providing continuous, high-resolution data on environmental and biological processes. They can be broadly classified into six major functional categories: optical, electromagnetic, electrochemical, location, acoustic, and airflow sensors [28] (Figure 2). Each type contributes unique data streams that, when integrated through IoT networks and analytical models, support precision decision-making and automated management.

#### 3.1.1. Optical Sensors

Optical sensors detect light intensity, chlorophyll fluorescence, crop coloration, and canopy reflectance, serving as critical indicators of plant health and photosynthetic efficiency [29]. These sensors are invaluable for early stress detection and can quantify the effects of microbial inoculants on chlorophyll content and canopy vigor.

#### 3.1.2. Electromagnetic Sensors

Electromagnetic sensors measure soil temperature, electrical conductivity, and water content, providing essential data for soil characterization and irrigation scheduling. This information helps calibrate microbial and nutrient applications according to site-specific field conditions, enhancing overall efficiency.

#### 3.1.3. Electrochemical Sensors

Electrochemical sensors quantify pH, nutrient ion concentrations, and vapor levels, offering accurate, real-time monitoring of soil and water chemistry. Their feedback supports the optimization of microbial survival, nutrient solubilization, and fertilizer-use efficiency.

#### 3.1.4. Biosensors for Soil Microbial Activity

Recent innovations extend electrochemical sensing to direct monitoring of microbial metabolism and soil biological activity. Impedance-based and microelectrode biosensors can quantify microbial respiration, redox potential, and enzyme activity in situ, providing real-time indicators of soil health and nutrient-cycling efficiency. For example, microfluidic–sensor platforms have successfully measured microbial redox fluxes and metabolic dynamics under field conditions, demonstrating their value for linking soil microbiome function with precision-farming analytics [30]. These biosensors bridge the gap between chemical sensing and biological assessment, integrating microbial ecology into smart-farming frameworks.

#### 3.1.5. Location Sensors (Geosensors)

Location sensors capture spatial coordinates and geospatial dynamics, which are essential for precision agriculture. Through GPS and geosensor networks, field variability and microbial inoculant performance can be effectively mapped and analyzed to support site-specific management [31].

#### 3.1.6. Acoustic and Airflow Sensors

Acoustic sensors detect vibrations and sound patterns linked to livestock health, insect activity, or mechanical operations, while airflow sensors measure temperature, humidity, and air circulation in greenhouses or livestock facilities. These devices contribute to climate control, animal welfare, and energy efficiency within integrated farming systems.

#### 3.1.7. Common Environmental Sensors in Agriculture

The most widely used environmental sensors include temperature, humidity, and soil-moisture sensors [22,32]:

Temperature sensors (thermistors, thermocouples, RTDs, infrared systems) offer high-accuracy monitoring, optimizing microbial inoculant viability and crop metabolic processes.

Humidity sensors (capacitive or resistive) track relative humidity, enabling preciseirrigation and greenhouse management.

Soil-moisture sensors provide real-time data on water availability, supporting data-driven irrigation scheduling that conserves water while maintaining ideal conditions for microbial activity and plant growth.

Today, most agricultural sensors operate wirelessly, transmitting data via Wi-Fi, Bluetooth, or LoRa networks to digital dashboards or mobile applications. These systems automate irrigation, fertilization, and inoculant delivery, significantly enhancing resource-use efficiency and environmental sustainability in modern smart-farming frameworks.

### 3.2. Data Collection and Analysis

#### 3.2.1. Data Acquisition Through On-the-Go Sensors

Modern on-the-go sensors enable continuous, high-resolution field data collection, capturing a wide range of environmental, soil, and crop parameters. Commercial systems such as Crop Circle (Holland Scientific, Lincoln, NE, USA), GreenSeeker (NTech Industries, Ukiah, CA, USA), CCS-645 and CCS-661 (Veris Technologies, Salina, KS, USA), SoilOptix (Soil Optix Inc., Tavistock, ON, Canada), Multiplex3 (Force A, Orsay, France), and RapidSCAN CS-45 (Holland Scientific, Lincoln, NE, USA) represent some of the most advanced solutions for real-time field assessment. Each platform differs in accuracy, cost, and maintenance requirements, allowing farmers to select systems best suited to local conditions, farm size, and resource availability. These sensors generate large datasets encompassing soil nutrients, canopy reflectance, and environmental variables, which form the foundation for real-time agronomic decision-making and precision resource management.

#### 3.2.2. Challenges of Interoperability and Data Standardization

The growing diversity of sensor brands, data formats, and communication protocols often creates interoperability challenges that hinder smooth information exchange across systems. File incompatibility, inconsistent metadata, and proprietary software restrictions complicate processing, storage, and transfer, particularly when third-party software cannot interpret closed-source formats [33]. This fragmentation weakens the traditional data “language” connecting users to their devices, resulting in inefficiencies in integrated farm management. Therefore, establishing interoperable data architectures and standardized exchange protocols is essential to ensure seamless workflow consistency across sensors, platforms, and farm operations.

#### 3.2.3. Farm Management and Decision Support Systems

Farm Management Information Systems (FMIS) have evolved to incorporate the analytical capabilities of Decision Support Systems (DSS). These integrated systems perform complex analyses that determine optimal dosages, timing, and spatial locations for the application of fertilizers, microbial inoculant, and irrigation water. Consequently, farmers increasingly serve as system supervisors who interpret and implement automated recommendations, rather than manually calculating management parameters [2]. While this shift enhances efficiency and precision, it also underscores the importance of user control, transparency, and data interpretability in algorithm-based agricultural management.

#### 3.2.4. Data Quality, Transfer, and Integration

High-quality data collection and error control are crucial for the accuracy and reliability of precision agriculture. Errors introduced during sensor calibration, data logging, or signal transmission can propagate through subsequent analyses, undermining the trustworthiness of decision-support outputs [31]. Efficient data-transfer frameworks must therefore enable automatic synchronization among multiple sensors and machines, ensuring traceability from raw measurements to processed outputs. Ideal systems provide cross-platform flexibility, preserve data provenance, and include Information Management System (IMS) tools for detecting and correcting missing or corrupted entries. Integrating satellite imagery with on-the-go soil sensor maps further enhances spatial resolution, allowing precise delineation of management zones and optimization of fertilizer or irrigation strategies [34]. Farms equipped with real-time sensors, robust data-handling infrastructures, and continuous quality control can transform raw data into actionable insights, ensuring precision, sustainability, and long-term productivity within the broader smart-agriculture ecosystem.

### 3.3. Integration with Farming Practices

A defining feature of smart agriculture systems is their adaptive capability to integrate diverse information sources and utilize the most relevant data for each management decision. In crop production, farmers can directly control three key parameters—timing, volume, and composition of water and nutrient inputs—to optimize plant growth and yield. Therefore, an effective digital agriculture platform must be built around a comprehensive soil–water–plant interaction model, calibrated to reflect local environmental and management conditions [35]. To achieve this, a wide range of data inputs is required. These include capacitance sensors for soil moisture measurement, fractional cover cameras for vegetation density estimation, and electrical resistivity tomography for characterizing soil texture and structure. In addition, advanced datasets such as satellite hyperspectral imagery, airborne gamma-ray surveys, and on-farm meteorological station data provide broader environmental context for model calibration and validation [36]. By combining these heterogeneous data layers, digital models can generate real-time state estimates, short-term forecasts, and predictive scenario, enhancing precision in irrigation scheduling, nutrient delivery, and microbial inoculant application. Emerging tools such as drone-based stereography, distributed image analysis, and machine-learning-driven data fusion can be seamlessly integrated to further expand predictive capacity and management precision [37]. Importantly, microbial data—including soil enzyme activity, respiration rates, and inoculant persistence-can also be incorporated into these models to refine recommendations for biofertilizer use and soil microbiome management. This integration transforms the digital platform into a Microbe–Sensor–Model feedback loop, ensuring adaptive nutrient cycling, early stress detection, and targeted interventions for improved sustainability. The information generated by these platforms is typically delivered to farmers through cloud-based interfaces and mobile applications, which present interactive dashboards summarizing soil water balance, nitrogen demand, microbial activity indices, and weather impacts. These systems allow for bidirectional interaction, where farmers can input local observations or field measurements that, in turn, refine model accuracy and enhance predictive reliability [4,18]. The combination of web accessibility, real-time alerts, and adaptive feedback mechanisms empowers farmers to make data-informed, location-specific decisions, effectively bridging the gap between technological sophistication and practical usability in the field.

## 4. Combining Microbial and Sensor Technologies

The integration of beneficial microbes with advanced sensor technologies creates a transformative framework for achieving sustainable and efficient crop production. In this combined approach, microbial inoculants enhance soil fertility, nutrient uptake, and plant vigor, while sensors provide continuous, real-time monitoring to optimize environmental conditions and resource inputs. Field-based studies have demonstrated the synergistic advantages of combining these innovations. For instance, a case study in England on winter wheat revealed that sensor-guided nitrogen management significantly increased the Critical Nitrate Concentration (CNC) associated with biomass growth—an effect largely attributed to microbial nitrate immobilization during early development. By applying nitrogen fertilizers at sensor-recommended intervals, farmers compensated for immobilization losses, achieving 11% higher yields compared with conventional practices [38]. Similarly, sensor-assisted micronutrient management has been shown to optimize nutrient uptake timing and maximize yield performance [39]. These examples confirm that integrating microbial functions with sensor-based control systems enhances both productivity and environmental sustainability. Together, microbial inoculants and precision-sensing technologies support the core objectives of Smart Agriculture, ensuring optimized nutrient cycles, reduced agrochemical dependency, and enhanced system resilience under climate and resource pressures [11,27,40].

### 4.1. Synergistic Effects

**Figure 3:**
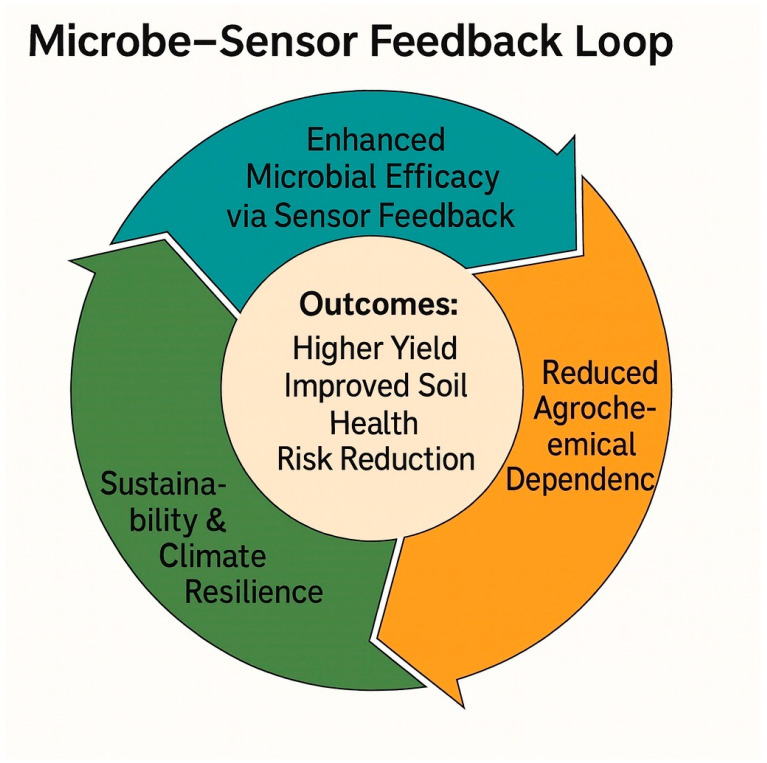
Circular framework illustrating the synergistic integration between beneficial microbes and sensor technologies in smart agriculture. The central Smart-MIP (Microbe–Information–Precision) feedback loop connects biological and digital systems, enabling real-time adjustment of microbial activity based on sensor-derived data. Arrows indicate continuous data flow between microbial performance monitoring (e.g., rhizosphere activity, enzyme secretion, nutrient solubilization) and sensor feedback on soil and crop status (e.g., pH, moisture, electrical conductivity, and plant stress indices). Three synergistic pathways—(1) enhanced microbial efficacy through optimized environmental conditions, (2) reduced agrochemical dependence by targeted microbial interventions, and (3) improved sustainability and climate resilience via adaptive management—jointly contribute to higher yields, improved soil health, risk reduction, and ecological balance. This framework highlights how integrating bio- and info-technologies can create a self-learning agricultural system for sustainable productivity.

The synergy between beneficial microbes and sensor systems can be classified into three major functional categories (Figure 3).

#### 4.1.1. Enhanced Microbial Efficacy Through Sensor Feedback

Beneficial microbes—including PGPR, rhizobia, arbuscular mycorrhizal fungi (AMF), and *Trichoderma* spp.—promote plant growth through nitrogen fixation, solubilization, siderophore production, phytohormone synthesis, and soil-structure improvement [6]. Sensor networks provide real-time data on soil moisture, pH, temperature, and nutrient concentrations, enabling precise adjustments to maintain optimal conditions for microbial survival and functionality. This continuous microbe–sensor feedback loop aligns biological activity with digital control systems, ensuring maximum efficacy of microbial inoculants.

#### 4.1.2. Reduced Dependence on Agrochemicals

Sensor-integrated microbial management reduces the need for synthetic fertilizers and pesticides by enabling data-driven decisions on the timing, dosing, and placement of bioinoculants and biocontrol agents [20]. Such optimization minimizes nutrient leaching, chemical runoff, and soil degradation, while enhancing soil biodiversity and long-term ecosystem health. This approach supports global sustainability goals and contributes to pollution mitigation through smarter, biologically driven input management.

#### 4.1.3. Support for Sustainable and Climate-Resilient Agriculture

The combined use of microbial technologies and sensor systems strengthens agroecosystem resilience under drought, salinity, and nutrient deficiency stress [10,11,18]. Smart-monitoring platforms equipped with IoT connectivity and predictive analytics enable the early detection of stress signals, allowing for timely microbial or irrigation interventions. The outcome is a Smart-MIP (Microbial- and Sensor-Based Integrated Precision) framework capable of sustaining high productivity while conserving energy, water, and nutrient resources.

### 4.2. Case Studies

The integration of microbial and sensor technologies has been validated globally through multiple case studies, demonstrating both economic feasibility and environmental sustainability. Across regions, microbial innovations have revolutionized crop management by enhancing nutrient efficiency, suppressing pathogens, and stabilizing yield. The following examples represent a combination of controlled experiments, pilot-scale validations, and commercial applications that collectively demonstrate how microbial technologies and smart-sensor systems function under different operational contexts. For example, in the banana industry of Asia (India and the Philippines) and East Africa (Uganda and Tanzania), productivity and plant health have been maintained through microbial inoculants, biocontrol agents, and microbial consortia that stimulate native soil microbiota and suppress pathogens [6]. In Brazil, large-scale adoption of nitrogen-fixing bacteria in soybean cultivation has reduced dependence on chemical fertilizers, confirming the economic and ecological viability of microbial technologies [41]. Likewise, co-inoculation with the mycorrhizal fungus *Gigaspora rosea* and the bioinoculant *Penicillium bilaiae* has improved phosphorus uptake and enhanced plant growth in soybean, wheat, and vineyard systems. In Venezuelan soils, combinations of phosphate-solubilizing bacteria (PSB) and plant growth-promoting rhizobacteria (PGPR) have improved nutritional status and yield stability in multiple crops. Parallel advances in sensor technologies provide the digital backbone supporting these microbial applications. Drone-mounted hyperspectral sensors enable high-resolution monitoring of soil and crop health, guiding targeted biopesticide application and resource allocation [40]. Companies such as Semios (USA) employ IoT-based wireless sensors to monitor microclimate conditions, insect activity, and plant health, improving pest management efficiency and reducing pesticide use. Similarly, AgriSens has developed portable, low-cost soil sensors that measure pH, moisture, and temperature, supporting data-driven microbial management at the field level. Collectively, these examples demonstrate the transformative power of microbe–sensor integration. The combination of biological inoculants and real-time sensor feedback enhances input efficiency, reduces environmental impact, and empowers farmers with actionable, site-specific information. Together, these examples demonstrate how the Smart-MIP (Microbial- and Sensor-Based Integrated Precision) approach can drive sustainable intensification and resilience in modern agriculture [42]. Representative examples of successful microbe–sensor integration across different crops and regions are summarized in Table 2. The practical applications and global case studies presented above highlight that integrating microbial and sensor technologies is not only a technological innovation but also a strategic foundation for sustainability. By combining real-time environmental monitoring with biologically driven nutrient and pest management, these systems reduce external chemical inputs, conserve natural resources, and enhance ecosystem resilience. Such integrative approaches bridge the gap between precision technology and ecological stewardship, directly leading to the sustainable practices discussed in the following section.

## 5. Sustainable Practices in Agriculture

Sustainability is one of the foremost priorities of 21st-century agriculture, emphasizing efficient resource utilization, environmental protection, and long-term food security. Within this framework, smart farming leverages intelligent monitoring systems and microbial-based technologies to produce only what is required for optimal yield and minimal waste. These digital-biological tools establish a self-regulating feedback loop that continuously adapts to environmental changes and production needs, thereby enhancing both productivity and resource efficiency [43]. A healthy and diverse soil microbiota forms the foundation of agricultural sustainability, maintaining nutrient cycling, soil structure, and resilience against biotic and abiotic stresses. However, excessive reliance on synthetic fertilizers and pesticides has disrupted these ecological functions, posing serious risks to soil fertility, ecosystem balance, and human health. Restoring sustainability requires the integration of four complementary strategies: (1) the application of beneficial microorganisms to stimulate plant growth and restore soil vitality, (2) bioremediation to detoxify environmental contaminants, (3) improved nutrient cycling through organic and microbial inputs, and (4) biological control to manage pests and diseases naturally [21]. The incorporation of sensor technologies further strengthens these strategies by providing real-time insights into soil moisture, nutrient availability, and microbial activity. This enables data-driven adjustments that minimize agrochemical inputs and optimize energy efficiency. The combined advancement of microbial inoculants, organic amendments, and precision-monitoring devices represents a clear pathway toward sustainable intensification—achieving global food security while safeguarding environmental integrity [10,18,20].

### 5.1. Case Studies in Sustainable Agricultural Practices

Sustainability in agriculture rests upon three interdependent pillars: environmental health, economic profitability, and social equity [44,45,46]. In recent years, smart farming approaches that integrate microbial biotechnologies with IoT-enabled sensing systems have demonstrated measurable progress across all three dimensions. For instance, microbial biofertilizers such as *Azospirillum* and *Rhizobium*, when applied to cereal and legume crops, have produced notable yield increases while reducing nitrogen fertilizer requirements by 30–40% in field-validated trials, thereby lowering production costs and mitigating greenhouse gas emissions [10]. Similarly, sensor-guided irrigation systems implemented in arid and semi-arid regions have optimized water use by up to 50% in commercial greenhouse and open-field systems, maintaining soil moisture levels that favor microbial colonization and nutrient exchange [18]. In India, where agriculture forms the economic backbone for millions, the combination of microbial inoculants with smart irrigation sensors has enhanced soil organic matter, reduced pesticide dependence, and increased both crop productivity and farmer income through large-scale implementation programs [10,18]. Likewise, in Mediterranean vineyard systems, the integration of arbuscular mycorrhizal fungi (AMF) inoculation with soil-nutrient sensors has significantly improved phosphorus uptake and mitigated eutrophication risk, as confirmed by pilot-scale field validations. Collectively, these examples illustrate how biological and digital innovations, when effectively combined, can reinforce ecological stability, strengthen resource efficiency, and promote economic and environmental sustainability simultaneously [10,18].

### 5.2. Benefits of Sustainable Agriculture

Sustainable agriculture encompasses a set of innovative practices designed to meet current and future food demands without compromising the health of ecosystems or the well-being of future generations. These approaches aim to protect the environment, enhance energy efficiency, and reduce ecosystem degradation, while maintaining economic viability and social equity [47]. Transitioning toward sustainability offers multidimensional benefits, extending beyond environmental preservation to include economic gains, social development, and technological innovation. The key outcomes of such systems include (1) enhanced soil fertility and microbial diversity, ensuring long-term productivity and resilient soil ecosystems; (2) reduced chemical input costs through the use of biofertilizers and precision nutrient management, guided by real-time sensor feedback; (3) improved community well-being through local employment opportunities, empowered farmer participation, and the establishment of resilient food systems; and (4) lower environmental footprint, achieved by minimizing soil erosion, nutrient leaching, and chemical pollution in agroecosystems [48,49]. Ultimately, the integration of renewable energy sources, beneficial microbial technologies, and sensor-driven decision support systems establishes a holistic smart-sustainability framework that aligns food production with environmental stewardship and climate resilience. This approach ensures that agricultural growth continues without exhausting the natural capital essential for future development [10,18].

## 6. Challenges in Implementing Smart Agriculture

Microbial technologies offer remarkable potential for transforming conventional agriculture into a sustainable and resilient system. Nevertheless, biological, technical, economic, and institutional barriers continue to limit large-scale adoption and integration with smart farming technologies. Biological limitations remain among the most critical obstacles. The high efficacy observed under controlled laboratory or greenhouse conditions often declines sharply under field conditions, where a complex interplay of soil type, climatic variability, and native microbial communities influences the performance of introduced strains [6,50,51]. Environmental parameters such as temperature fluctuations, pH changes, salinity, and pollutant presence frequently alter microbial survival and metabolic activity, resulting in inconsistent crop responses. Beyond biological challenges, technical and socioeconomic constraints also hinder broader adoption. Many bioformulations suffer from short shelf life, storage sensitivity, and incompatibility with commonly used agrochemicals or application equipment. These weaknesses are compounded by logistical difficulties, including inadequate cold-chain infrastructure and limited access to quality-control laboratories. Similarly, the implementation of advanced sensor technologies faces multiple challenges, such as high initial costs, regular calibration requirements, maintenance demands, and the lack of standardized data protocols across devices and regions [52,53]. Despite these hurdles, recent advances in Internet of Things (IoT) devices, embedded sensors, and ubiquitous sensor networks are beginning to bridge the technology gap. The development of affordable, low-power sensor–actuator platforms with open communication standards now allows precise environmental control in greenhouse and hydroponic systems. These innovations demonstrate that cost-effective and user-friendly digital tools can significantly improve confidence in precision agriculture, while simultaneously reducing energy consumption and input waste [11,54]. The key categories of challenges and their corresponding mitigation strategies are summarized in Table 3, providing a concise overview of the main constraints and potential solutions for the scalable implementation of sustainable smart agriculture.

Furthermore, although microbial and sensor-based technologies show promising results under laboratory and greenhouse conditions, their field performance frequently declines under variable environmental contexts. Typical field-to-laboratory yield gaps range from 15% and 25%, reflecting variability in soil structure, climatic conditions, and microbial interactions with indigenous communities. From an economic perspective, microbial inoculant formulations generally cost USD 20–50 per hectare, whereas integrated sensor systems—including IoT infrastructure and maintenance—range between USD 500 and USD 2000 per hectare, depending on the scale and technical sophistication. These performance and cost gaps highlight the need for adaptive calibration, localized field validation, and cost-sharing frameworks to promote equitable and widespread adoption of smart-agriculture innovations [50,52,53,54].

### 6.1. Technical Barriers

The integration of beneficial microbes with sensor-based systems offers tremendous potential to enhance the economic and environmental sustainability of modern agriculture. However, technical limitations remain among the most persistent obstacles to the effective realization of this synergy. Limited mechanistic understanding continues to restrict large-scale deployment. Despite significant advances in molecular biology and microbial ecology, only a few model microbial species have been studied in sufficient detail to elucidate their interactions with host plants and environmental variables [50]. As a result, the translation of laboratory discoveries into reliable field applications remains slow and inconsistent. Technology readiness and scalability issues further complicate progress. Although next-generation sequencing (NGS), genetic engineering, and nanoparticle-assisted delivery systems are advancing rapidly, their adaptation for on-farm use remains limited by high costs and a lack of standardized protocols [55]. Consequently, most commercial microbial inoculants continue to rely on native or naturally occurring strains, since genetically engineered microbes have not yet demonstrated consistent field performance or achieved broad regulatory acceptance. The use of indigenous microbial consortia remains the most viable and ecologically compatible strategy, as it supports local adaptation and fosters farmer acceptance. Integrating these native microbial communities with sensor-driven monitoring platforms can substantially improve efficiency by providing real-time feedback on nutrient availability, soil moisture, and microbial activity. Furthermore, nanotechnology is emerging as a powerful complementary tool, enabling development of targeted nutrient formulations and protective carriers that enhance microbial survival and functionality under variable environmental conditions [15]. Collectively, these technical innovations form a strong foundation for overcoming existing challenges and achieving scalable, precision-based microbial applications that reinforce the sustainability goals of smart agriculture.

### 6.2. Economic Barriers

Despite the well-documented benefits of microbial technologies and sensor-based systems, economic limitations remain a major constraint to their widespread implementation. The high initial investment costs for sensors, IoT infrastructure, and precision-monitoring devices continue to discourage many small- and medium-scale farmers from adopting these innovations. Similarly, the production and commercialization of high-quality microbial inoculants require specialized facilities, controlled fermentation systems, and stringent quality assurance, all of which increase production costs and limit market accessibility [52]. In many developing regions, farmers tend to prioritize short-term cost savings over long-term ecological sustainability, largely due to restricted access to credit, limited government incentives, and insufficient financial literacy. The absence of targeted subsidy programs or cooperative procurement models further intensifies affordability issues. Conversely, large agribusinesses in developed economies often dominate adoption because they can offset investment costs through economies of scale and higher profit margins [52,54]. To mitigate these challenges, targeted financial mechanisms—such as microcredit schemes, public–private partnerships (PPPs), and tax incentives for sustainable agricultural inputs—are urgently required. Additionally, cooperative-based equipment-sharing models, combined with low-cost IoT sensor platforms, can significantly reduce per-unit expenses and increase accessibility for smallholder farmers. Implementing such inclusive financial frameworks would not only improve adoption rates but also promote equitable participation across smart agriculture value chains, ensuring that technological innovations in microbial and sensor systems benefit farmers at all socioeconomic levels [11,54].

### 6.3. Regulatory and Social Barriers

In addition to technical and economic challenges, the regulatory and social dimensions of smart agriculture present significant barriers to large-scale adoption. Regulatory frameworks governing microbial inoculants, genetically modified organisms (GMOs), and IoT-enabled devices remain fragmented and inconsistent across regions, leading to uncertainty for producers, distributors, and end-users. In many countries, standardized procedures for efficacy testing, biosafety evaluation and product certification are lacking, resulting in variable quality assurance and reduced market confidence [52]. Social factors are equally important in determining adoption success. Farmer reliance on traditional agrochemical practices, limited awareness of microbial and digital innovations, and concerns about data privacy in IoT systems collectively contribute to low acceptance rates. Additionally, disparities in digital literacy—especially among smallholders, older farmers, and rural communities—further limit participation in technology-driven initiatives. To address these challenges, policy harmonization and the development of transparent, science-based guidelines for product registration, biosafety assessment, and data management are essential. Establishing national certification systems and extension programs would strengthen market trust and ensure consistent regulatory oversight. On the social side, capacity-building initiatives, including demonstration farms, hands-on training, and participatory workshops, can enhance awareness and build farmer confidence in both microbial and digital technologies. Bridging the gap between scientific research, policy development, and community engagement will accelerate the social integration of smart agriculture and ensure that its benefits are distributed equitably across different farming systems and socioeconomic contexts [11,52,54].

## 7. Future Trends in Smart Agriculture

**Figure 4:**
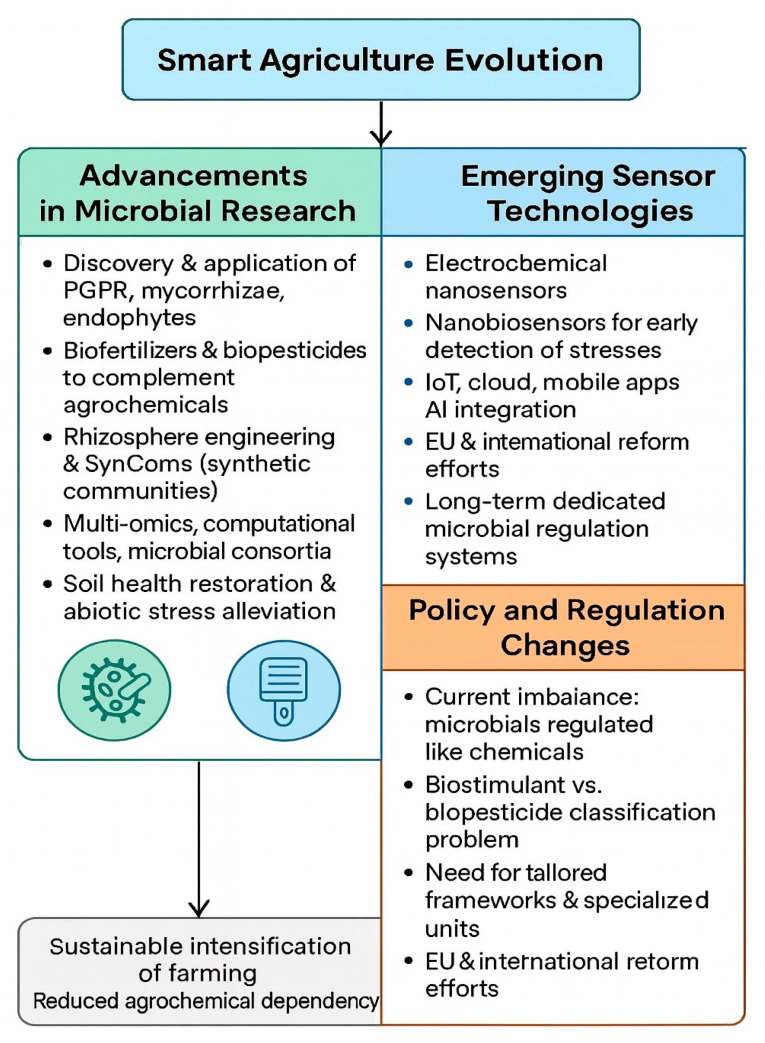
Flow schematic summarizing the future trends and transformative directions in smart agriculture. The diagram highlights three core pillars driving this evolution: (1) advancements in microbial research, focusing on the development of multifunctional biofertilizers, biostimulants, and biocontrol agents that enhance plant productivity and resilience; (2) emerging sensor technologies, including next-generation IoT devices, remote sensing platforms, and machine-learning-driven data analytics for real-time monitoring and adaptive management; and (3) evolving policy and regulatory frameworks, aimed at harmonizing biosafety standards, encouraging sustainable practices, and promoting innovation in agri-biotechnology. Arrows indicate the flow of innovation and feedback among these domains, showing how scientific, technological, and regulatory progress converge to reduce agrochemical dependency, restore soil health, improve climate resilience, and ensure sustainable intensification of farming systems.

Smart agriculture is undergoing rapid transformation through the integration of plant-growth-promoting microbes (PGPMs), sensor-based nutrient management systems, and digital automation frameworks that collectively enhance productivity, efficiency, and sustainability (Figure 4). Intensive research on plant-protective microorganisms, including biofertilizers and biopesticides, continues to reduce dependence on synthetic agrochemicals and improve soil health [6]. At the same time, advances in nanotechnology and biotechnology are revolutionizing the formulation, delivery, and field performance of microbial inoculants. The development of encapsulated or nano-assisted microbial carriers improves stability, shelf life, and targeted release, enabling more consistent in-field performance under diverse environmental conditions [15]. Emerging research is also focused on microbes that alleviate abiotic stress—such as salinity, drought, and heavy metal toxicity—and those that restore soil structure and nutrient cycling, establishing a new frontier for climate-resilient agriculture [20]. Parallel to these biological innovations, the incorporation of Internet-of-Things (IoT) devices, cloud-based analytics, mobile applications, and Web-of-Things (WoT) platforms is driving the transition toward autonomous, data-driven, and self-regulating agricultural systems. Looking ahead, the next generation of smart agriculture will be defined by AI-assisted microbial modeling, which will enable the simulation and prediction of complex microbe–plant–soil interactions under dynamic environmental conditions. These predictive models, coupled with machine-learning algorithms, will support the design of optimized microbial consortia and adaptive management strategies. The proliferation of low-cost, open-source IoT sensor networks will further democratize access to precision agriculture, particularly for smallholder and resource-limited farmers. Meanwhile, bio-digital twin technologies—virtual models integrating microbial genomics, soil chemistry, and environmental data—will provide powerful platforms for decision-making and sustainable field management. Future research should prioritize data interoperability and integration between biological and environmental datasets, ensuring seamless communication between microbial monitoring, sensor systems, and AI analytics. Additionally, advancements in nanobiosensors, wearable plant sensors, and remote microbial-sensing systems will enable early stress detection and real-time ecosystem diagnostics. Finally, updated policy and regulatory frameworks will be essential to govern data sharing, biosafety, and ethical technology deployment. Additionally, future microbial applications should consider the balance between universal inoculant formulations and locally adapted microbial consortia. While standardized bioformulations offer scalability and ease of quality control, their efficacy often varies under different soil, climatic, and cropping conditions. Leveraging native microbiomes and region-specific strains can improve colonization success, persistence, and ecological compatibility with local environments. Integrating such site-tailored approaches into AI-driven microbial modeling and prediction frameworks may further enhance precision, resilience, and sustainability across diverse agroecosystems. Collectively, these technological and research trends demonstrate that the future of agriculture lies in the synergistic fusion of biological intelligence and digital precision. This microbe–sensor–data integration will not only reduce agrochemical dependency and restore ecosystem balance, but also enhance climate resilience, optimize productivity, and ensure long-term food security through sustainable intensification.

### 7.1. Advancements in Microbial Research

Future microbial research will focus on characterizing beneficial organisms, deciphering their interactions and mechanisms, and designing synthetic microbial communities (SynComs) with greater field reliability. Although microbial inoculants have been used for over a century, their large-scale success has only recently become achievable through multi-omics and computational advances [50]. Biofertilizers and biopesticides are increasingly regarded as complements rather than replacements for chemical fertilizers and pesticides, thereby aligning with integrated crop management strategies [11]. Their effectiveness is typically evaluated through increased yields, reduced chemical inputs, and demonstrable environmental benefits. In developing regions, microbial inoculants also promote local production, generate employment opportunities, and strengthen regional sustainability, making them valuable socio-economic tools. To ensure consistent efficacy, collaborative efforts among research institutions, policymakers, industry stakeholders, and farmers are essential. Public–private partnerships and long-term research commitments can facilitate improvements in formulation technologies, extend product shelf life, and optimize delivery mechanisms. Artificial intelligence (AI) and data-driven modeling are emerging as transformative tools for both microbial research and application. Recent frameworks integrate genomic, soil, and climatic data to predict optimal microbial inoculants and application timings for specific crops and environments. For example, an AI-assisted recommendation system can analyze microbial community profiles and edaphic factors to suggest compatible microbial consortia that enhance nutrient use efficiency and stress resilience. When coupled with sensor feedback, these predictive tools can dynamically adjust inoculation strategies based on real-time field data, offering site-specific, evidence-based solutions that enhance both microbial performance and sustainability [56]. Rising food demand, dietary transitions, and soil degradation necessitate intensified yet sustainable production systems. Manipulating the rhizosphere microbiome and promoting soil-organic-matter accumulation are key to improve plant resilience and nutrient efficiency. Management tools such as microbial inoculation, crop rotation, and optimized irrigation scheduling remain central to maintaining soil health [18]. Technological advances in metagenomics and multi-omics have greatly expanded our understanding of the soil microbiome, yet important questions remain regarding how tillage practices and cropping systems influence microbial community structure and function. Addressing these knowledge gaps will accelerate the design of SynComs, engineered microbial consortia capable of enhancing plant stress tolerance and boosting productivity [57].

### 7.2. Emerging Sensor Technologies

The new era of Smart Agriculture 4.0 demands robust, real-time monitoring tools that effectively link biological and environmental data. Electrochemical and nanobiosensors, empowered by nano-electrochemistry, have become central components of precision farming, enabling rapid, low-cost, and highly sensitive measurements of soil, plant, and environmental parameters [27]. These technologies facilitate the early detection of nutrients, metabolites, pesticides, pathogens, and stress biomarkers, allowing interventions before significant crop losses occur. Nanoscale electrodes provide a larger surface-to-volume ratios compared to conventional sensors, thereby enhancing signal sensitivity and response speed. When combined with smartphone interfaces, they permit in situ, user-friendly and cost-effective field operation. In parallel, nanoparticle-based agrochemical formulations—including nano-fertilizers, nano-herbicides, and nano-fungicides—enable targeted and efficient delivery, significantly reducing environmental contamination and resource waste [32]. Digital connectivity, supported by IoT platforms, and cloud analytics is transforming traditional agriculture into a data- and knowledge-driven ecosystem. These technologies empower producers to optimize inputs, reduce waste, enhance traceability, and strengthen consumer trust by improving transparency throughout the supply chain [58]. Moving forward, validation under field conditions and comprehensive farmer education will be crucial to ensure practical implementation. The successful adoption of emerging sensor technologies will depend on hands-on training programs, demonstration projects, and data-sharing frameworks that connect farmers, researchers, and policymakers, fostering collaboration and sustainable innovation [23].

### 7.3. Policy and Regulation Changes

Policy and regulatory frameworks are increasingly adapting to the expanding role of microorganisms in crop protection and plant growth promotion. Many microbial species perform dual functions—acting simultaneously as biostimulants and biopesticides—yet current regulations often prohibit dual marketing, reflecting a lack of clear taxonomic and functional classification [59]. Existing authorization systems were primarily designed for chemical pesticides, making them poorly suited to the unique biological and ecological characteristics of microbial agents. The absence of dedicated legislation results in regulatory imbalance, lengthy approval procedures, and restricted commercialization [60]. To address these challenges, the establishment of microbial-specific regulatory frameworks or specialized divisions within existing systems would help streamline product registration, ensure biosafety, and encourage scientific and industrial innovation [61]. Efforts in the European Union and other regions are currently focused on harmonizing and expediting approval processes, but achieving comprehensive legislative reform remains a long-term policy goal. Any future regulatory frameworks should include well-defined data requirements, evaluation standards, expertise criteria, and institutional responsibilities [62]. Such reforms are essential to promote the safe, transparent, and science-based implementation of microbial and sensor technologies worldwide, ensuring that innovation proceeds in balance with biosafety and sustainability objectives.

## 8. Case Studies of Successful Implementations

The practical integration of beneficial microbes with advanced sensor systems demonstrates the tangible potential of smart and sustainable agriculture. Microbiome-based innovations have already led to commercialization successes, yield improvement, disease resistance, improved nutrient uptake, drought tolerance, and even advances in food-safety monitoring and regulation. Several successful case studies highlight this synergy between biotechnology and digital technologies. The bacterial species *Sphingomonas melonis* has been shown to enhance disease resistance in rice, thereby reducing reliance on chemical fungicides. Likewise, nitrogen-fixing bacteria have replaced substantial portions of chemical fertilizers in Brazilian soybean production, offering both economic and ecological advantages. Fungal bioinoculants, such as *Penicillium bilaiae*, have improved phosphorus solubilization and availability across multiple cropping systems, while *Bacillus simplex* has been demonstrated to promote maize drought tolerance under water-limited conditions. In parallel, Internet-of-Things (IoT)-based multi-sensor systems have revolutionized farm management by enabling real-time soil and environmental diagnostics. These sensor networks continuously collect data on soil moisture, nutrient availability, and microclimate conditions, which can be integrated with microbial inoculation strategies to optimize resource use. When combined with precision-agriculture algorithms, such systems increase fertilizer efficiency, improve irrigation scheduling, and enhance pest management, while simultaneously minimizing environmental contamination. Despite these technological advancements, adoption remains limited in many regions, particularly where traditional manual practices still dominate. The integration of sensors and IoT systems into microbial-assisted farming establishes dynamic feedback loops that connect plant, soil, and environmental data. This integrated approach enables optimized fertilizer application, refined machinery routing, and the implementation of decision-support platforms that link plants, animals, equipment, and facilities through wired or wireless networks. The resulting applications include environmental monitoring, water-saving irrigation, greenhouse-gas mitigation, weather forecasting, food safety assurance, product traceability, and smart equipment management. Collectively, these innovations advance the integration of historical datasets and real-time information, enabling the development of accurate predictive models and sustainable agricultural solutions [4,42].

### 8.1. Global Examples

To meet the rapidly increasing global demand for agricultural productivity while ensuring the conservation of natural resources, many regions are adopting microbial- and sensor-based sustainable practices. Over the past decade, international organizations such as the World Bank, FAO, IFAD, and the World Wildlife Fund have actively promoted climate-smart agricultural technologies that integrate beneficial microbes with digital and sensor systems thereby improving adaptation to changing environments and emerging stress factors [18]. In Brazil, the soybean industry has demonstrated the effectiveness of nitrogen-fixing bacteria in reducing fertilizer input while maintaining high yields, serving as a model for sustainable intensification [41]. Similarly, *Penicillium bilaiae* bioinoculants, when combined with precision-sensor guidance, have significantly enhanced phosphorus-use efficiency in vineyards and wheat systems, particularly in nutrient-deficient soils. In Asia, *Sphingomonas melonis* has been successfully applied to improve disease resistance in rice, representing a major breakthrough in pathogen-prone ecosystems (Table 4). Sensor-driven strategies complement these microbial applications by enabling adaptive, data-informed management. In Europe and the United States, IoT-based soil-moisture and nutrient sensors are increasingly integrated with microbial biostimulant programs, allowing real-time nutrient adjustment and reducing chemical dependency. Furthermore, predictive climate models linked to sensor networks now assist in critical agronomic decisions, including genotype selection, planting time optimization, and stress mitigation under drought and heat conditions. Early-season growth regulation through plant-growth-promoting microbes, combined with precise environmental monitoring, has been shown to reduce terminal water-stress damage and stabilize yields under variable climatic conditions, providing a robust pathway toward resilient and sustainable global agriculture [11].

### 8.2. Local Innovations

At the national and community levels, a growing number of localized innovations illustrate how microbial and sensor-based technologies can simultaneously improve livelihoods and enhance sustainability. In Pakistan, the adoption of smart-agriculture systems has led to substantial improvements in water- and nitrogen-use efficiency, while also promoting novel, resource-efficient cultivation practices [11]. Saffron production, a high-value crop, serves as a notable example of these benefits—integrating beneficial microbes, IoT-based irrigation, and solar-powered monitoring systems to address food and nutritional insecurity, while empowering rural women and youth through participation in sustainable production networks. The establishment of microfinance programs for peasant and smallholder farmers has further facilitated access to essential technologies, including climate-resilient seeds, energy-efficient solar pumps, and drip-irrigation systems. In parallel, small-scale and rooftop renewable-energy installations are providing affordable electricity for smart irrigation, sensor networks, and cold-chain management, enabling farmers to reduce post-harvest losses and improve market access. These regional initiatives highlight that successful implementation of sustainable agricultural practices requires more than technological innovation alone—it depends on supportive policy frameworks, capacity-building programs, and interdisciplinary collaboration among biologists, engineers, agronomists, and policymakers. Collectively, these efforts demonstrate that the integration of beneficial microbes with advanced sensing technologies can foster inclusive, climate-resilient, and economically viable agricultural systems that safeguard both productivity and ecosystem integrity [11].

## 9. Economic Impacts of Smart Agriculture

**Figure 5:**
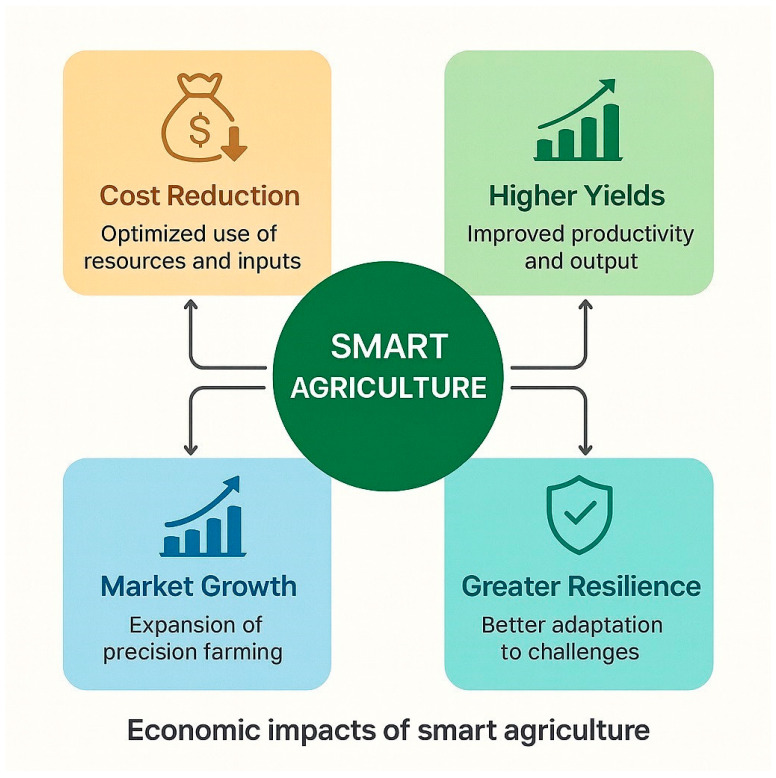
Economic and market impacts of microbe–sensor integration in smart agriculture. The schematic illustrates how the synergistic interaction between beneficial microbial inoculants (e.g., biofertilizers, biostimulants, and biocontrol agents) and sensor-based precision systems (monitoring soil fertility, crop growth, and environmental parameters) contributes to both economic efficiency and market development. Arrows and loops depict a positive feedback cycle in which cost reductions—achieved through optimized input use and higher productivity—stimulate technology adoption, market reinvestment, and agri-tech innovation. This integration promotes long-term profitability for farmers, encourages sustainable business models, and accelerates the transition toward environmentally responsible production. The figure emphasizes that the microbe–sensor nexus not only enhances resource efficiency and yield stability, but also drives global market growth and competitiveness within the agricultural technology sector.

The implementation of smart agriculture has produced measurable economic benefits through enhanced productivity, resource efficiency, and input optimization across the entire agricultural value chain. By integrating Internet-of-Things (IoT) technologies, sensor-based precision systems, and beneficial microbial applications, farmers can now apply fertilizers, pesticides, and irrigation water with unprecedented precision, responding dynamically to real-time field conditions [2]. This targeted management approach minimizes resource waste, reduces input costs, and often increases crop yields, demonstrating a direct return on technological investment. Beneficial microbes—long recognized for their plant-growth-promoting capacities—have gained renewed attention as technological innovations have improved their isolation, cultivation, and commercial-scale delivery. Agriculture is now emerging as a key application domain for synthetic biology and microbial biotechnology, in which engineered or naturally optimized microbial strains are developed and applied as biofertilizers, biostimulants, and biocontrol agents [15]. Once identified, characterized, and mass-produced, these microbial products become valuable biological resources that can either be integrated into existing production systems or marketed as sustainable agricultural inputs, expanding both economic opportunity and technological innovation within the bioeconomy. The economic potential of microbe-enhanced smart farming is particularly significant under conditions of climate variability. By improving nutrient-use efficiency, water optimization, and stress tolerance, microbial inoculants reduce the reliance on costly chemical inputs and mitigate yield losses caused by drought or salinity. When paired with sensor technologies, these biological interventions enable continuous monitoring of soil fertility, crop health, and environmental parameters, effectively transforming high-throughput data into actionable field-level insights. This integration creates a closed feedback loop that supports adaptive, data-driven management, leading to a more profitable and resilient agricultural economy. Documented outcomes include reductions in fertilizer and pesticide expenditure, yield increases of up to 10–20% in microbial-assisted systems, and lower long-term costs associated with environmental remediation. Beyond the farm level, the adoption of smart-agriculture technologies stimulates rural employment through technology manufacturing, data analytics, and microbial inoculant production, thereby strengthening local economies. Collectively, the integration of microbial biotechnology and precision sensing represents the culmination of a century-long innovation trajectory that reinforces agricultural sustainability, ensures economic viability, and enhances climate resilience (Figure 5).

### 9.1. Cost–Benefit Analysis

Achieving sustainable increases in global food production requires strategies that simultaneously address supply inconsistencies and inefficiencies across the agri-food value chain, while mitigating biodiversity loss, carbon emissions, and environmental degradation. Traditional intensification approaches—typically dependent on heavy agrochemical inputs—are no longer economically feasible or ecologically sustainable. In contrast, the integration of beneficial microbes with smart sensor technologies provides a viable solution for improving the cost–benefit ratio of food production by enhancing efficiency and reducing waste across the entire agricultural system [48]. Microbial inoculants contribute significantly to this balance by improving nutrient uptake, enhancing soil structure, and increasing plant stress tolerance, thereby reducing fertilizer and pesticide expenditures. When coupled with IoT-based precision management systems, which optimize water and nutrient delivery through real-time feedback loops, farmers can achieve higher yields at lower input costs. Collectively, these innovations result in tangible economic benefits—commonly a 10–30% reduction in input costs and a 10–20% improvement in yield efficiency, depending on crop type and environmental conditions. These improvements strengthen both farm profitability and resilience, while contributing to broader sustainability objectives. Beyond primary production, there is an increasing global demand for agricultural and food products that extend beyond basic nutrition to provide added health-promoting and sensory qualities. In this context, microbially influenced manufacturing is emerging as a key innovation area. This approach enables the stabilization and enhancement of natural flavors, the biosynthesis of nutraceutical compounds, and the biotransformation of raw agricultural materials into safer, more functional, and desirable food products [63]. Moreover, these microbial processes enhance product safety, consistency, and biodegradability, thereby reducing environmental impact and building consumer confidence [10,11,64]. Ultimately, the integration of microbial biotechnology with sensor-based precision systems establishes a positive feedback economy, where real-time data enhances microbial performance, and microbial activity in turn improves the precision and profitability of farming decisions. This circular and self-reinforcing model promotes long-term cost savings, reduces risk exposure, and supports the development of a sustainable agri-tech economy that effectively balances productivity with ecological stewardship.

### 9.2. Market Trends

The global market for precision and smart agriculture has expanded rapidly in recent years, reflecting the growing adoption of digital technologies, microbial innovations, and sustainability-oriented solutions. Valued at approximately USD 7.0 billion in 2021, the precision farming market is projected to reach USD 20.9 billion by 2026, representing an annual growth rate exceeding 19% and signaling its transition from a developing to a mature global industry [11]. Over the past two decades, advances in sensor miniaturization, cloud-based data management, and IoT connectivity have transformed agriculture into a data-driven enterprise, capable of integrating biological and technological systems in real time. In the United States, nearly 80% of farmers now utilize smart devices—including satellite-based monitoring tools, drones, and in-field sensors—to optimize water and nutrient use, detect crop stress, and reduce agrochemical dependence. This widespread digital adoption underscores the market’s increasing confidence in technology-enabled decision-making and its demonstrated financial returns. Emerging economies are also becoming key drivers of this digital transformation. For instance, Vietnam’s smart-agriculture market is projected to grow from USD 135 billion in 2023 to nearly USD 205 billion by 2024, achieving an 8.0% compound annual growth rate (CAGR) [10]. This rapid expansion reflects strategic national investments in telecommunications infrastructure, sensor manufacturing, semiconductors development, optoelectronics, and data services, all of which collectively underpin modern smart-farming systems. Market growth is driven not only by digital technologies but also by advancements in microbial biotechnology, which complement precision systems by improving crop yield, soil health, and resource-use efficiency. As modern farms increasingly integrate sensor-based nutrient monitoring, GPS-enabled machinery, and cloud-linked microbial management platforms, the synergy between living biological systems and computational technologies is emerging as a defining characteristic of the modern agricultural economy. Together, these innovations guarantee higher productivity, improved crop quality, and more sustainable agricultural value chains, establishing smart agriculture as a cornerstone of future global food security.

## 10. Environmental Impacts of Smart Agriculture

Plant growth and crop productivity are deeply interconnected with the renewal, diversity, and metabolic activity of beneficial microbial communities inhabiting the rhizosphere. This dynamic soil ecosystem hosts a wide range of plant-growth-promoting bacteria (PGPB) such as *Rhizobium*, *Pseudomonas*, *Bacillus*, *Azotobacter*, *Azospirillum*, and *Frankia*, along with arbuscular mycorrhizal fungi (AMF) belonging primarily to the genus *Glomus*. These microorganisms exert profound effects on plant performance by facilitating nutrient acquisition, improving root architecture, enhancing stress tolerance, and stimulating phytohormone production. Collectively, their synergistic actions lead to substantial yield improvements in major crops such as rice, corn, and wheat, as well as fruits and vegetables [6]. Beyond their role in productivity, beneficial microbes are indispensable for maintaining soil fertility and ecological balance. They accelerate nutrient cycling, improve soil aggregation and structure, and promote carbon sequestration, thereby contributing to long-term soil health. The integration of sensor technologies into microbially enriched agricultural systems marks a significant step forward in achieving environmental sustainability. Real-time sensor monitoring allows for precise quantification of soil nutrient status, moisture dynamics, and microbial activity, facilitating efficient nutrient delivery and optimized irrigation management [20]. When integrated into smart agricultural frameworks, these technologies reduce chemical fertilizer dependency, minimize nutrient leaching, and lower greenhouse gas emissions, mitigating agriculture’s contribution to climate change. This synergistic approach between beneficial microbes and sensor-based precision management directly supports global sustainability objectives, advancing environmentally responsible production systems that safeguard both soil and water resources. Ultimately, the integration of microbial biotechnology with digital sensing and IoT-based precision tools represents an effective strategy for optimizing resource use, enhancing ecosystem resilience, and minimizing agriculture’s environmental footprint [65].

### 10.1. Soil Health Improvement

Soil health represents the sustained capacity of soil to function as a vital living ecosystem that supports plants, animals, and humans alike. Within the framework of smart agriculture, the integration of advanced technologies, beneficial microbial inoculants, and digital monitoring platforms plays a decisive role in promoting soil sustainability and long-term productivity. The deterioration of soil health remains a multifaceted global challenge, both difficult and costly to reverse, often requiring several decades- or even centuries- for full recovery. Consequently, proactive protection and adaptive management of soil ecosystems are essential to ensure agricultural resilience and ecosystem stability [66]. Among the most effective strategies for improving soil health is the application of beneficial microbes, which maintain the dynamic biological processes underlying nutrient cycling, organic matter decomposition, and soil structural integrity. These microbes foster soil aggregation, improve nutrient bioavailability, and suppress soil-borne pathogens, thereby strengthening the soil’s natural fertility. Their symbiotic and associative interactions with plant roots enhance rhizosphere activity, stimulate root exudation, and promote the development of a self-sustaining microbial network that supports both soil functionality and crop performance. However, threats to soil health have intensified due to unsustainable farming practices, overreliance on chemical inputs, increased population pressures, and the adverse impacts of climate change. Soil acidification, organic carbon loss, and declines in microbial and faunal biodiversity are becoming increasingly widespread. In this context, the integration of beneficial microbial technologies with conventional agronomic methods has proven highly effective in improving soil productivity, stability, and resilience. This synergistic approach creates a biologically active and chemically balanced soil environment, resulting in enhanced crop yields, reduced input dependency, and long-term ecosystem sustainability [67,68,69].

### 10.2. Biodiversity Enhancement

Smart agriculture encompasses a wide range of technological innovations strategically designed to improve farming efficiency while maintaining environmental integrity and supporting biodiversity. Within this framework, beneficial microbes play a central role by enabling plants to fully express their genetic potential. Through complex biochemical and molecular interactions, these microbes modulate plant growth and development, enhance defense mechanisms against pests and pathogens, and improve overall plant resilience and productivity under variable environmental conditions [70]. In parallel, advanced sensor technologies provide real-time monitoring of both crop and environmental parameters, establishing a feedback system that supports adaptive management and evidence-based decision-making. For example, precision devices, such as innovative sunflower sensors, can accurately detect water stress in crops, providing farmers with critical data for optimizing irrigation scheduling and resource allocation [71]. The integration of biological and digital systems not only enhances plant performance but also preserves ecosystem stability. The synergy between microbial inoculants and sensor-based technologies fosters a form of biodiversity-oriented smart agriculture. Beneficial microbes enrich soil biodiversity and nutrient cycling, while smart sensors help minimize the overuse of water and agrochemicals, thereby reducing environmental contamination and protecting non-target organisms. Collectively, these practices support functional biodiversity, ensuring that diverse organisms—from soil microorganisms to pollinators—coexist in ecological balance within agroecosystems. From a broader sustainability perspective, this integrated approach aligns with the One Health concept, which recognizes the interconnectedness of human, animal, plant, and environmental health. The combined use of bio-nanofertilizers, bio-nanopesticides, co-cropping systems, and precision-farming technologies further amplifies these benefits. The integration of traditional ecological knowledge with modern technological innovation thus promotes a holistic and resilient agricultural paradigm, capable of enhancing productivity, conserving biodiversity, and sustaining the ecological equilibrium for future generations [50].

## 11. Social Implications of Smart Agriculture

Smart agriculture represents a transformative opportunity to move beyond conventional intensive farming systems that have historically depleted natural resources and weakened the resilience of agroecosystems. By integrating distributed sensor networks, wireless communication systems, cloud-based analytics, big data, and artificial intelligence, smart agriculture enables continuous and precise monitoring of crops, soil, and environmental variables. This data-driven framework empowers farmers to make informed management decisions, reduce resource wastage, and optimize crop performance, thereby improving both environmental and socioeconomic outcomes [10,11,18,20]. The incorporation of beneficial microbes further enhances the social and ecological value of these technologies. Microbial inoculants contribute to increased crop productivity, greater nutrient and water-use efficiency, and improved soil health, making them essential components for promoting sustainability and food security in modern agriculture. When integrated with sensor technologies, these biological innovations support the development of dynamic models describing microbe–plant–environment interactions, allowing for real-time tracking of microbial performance and ecosystem responses. For instance, because drought and nitrogen starvation can significantly reduce microbial efficiency, sensor-derived data can inform farmers—through simple algorithms—of the optimal timing and location for microbial application, ensuring maximum benefits under specific environmental conditions. Such sensor-guided microbial management has been shown to result in yield increase of 8.5–11.5% in maize and tomato compared to untreated fields [11,18,22]. Beyond direct productivity gains, smart agriculture carries profound social implications. It promotes sustainable livelihoods by reducing input costs, enhancing resource efficiency, and increasing resilience to climate variability. The widespread availability of affordable sensors and microbial products also supports smallholder inclusion, effectively bridging the technological gap between resource-rich and resource-limited regions. Moreover, digital platforms and IoT-based agricultural applications facilitate knowledge sharing, data democratization, and participatory decision-making, empowering farmers—especially women and youth—with access to actionable information and modern production tools [48,56]. Smart agriculture further contributes to rural community revitalization by creating employment opportunities in data analysis, sensor installation and maintenance, microbial inoculant production, and sustainable supply chains management. Collectively, these innovations promote a transition toward socially equitable, environmentally conscious, and economically viable farming systems, reinforcing the vision of “technology for people and planet.” As a result, the integration of beneficial microbes with sensor-based precision management not only improves yields and resource efficiency but also establishes social sustainability as a core pillar of the smart agriculture paradigm.

### 11.1. Community Engagement

Community engagement is a cornerstone of sustainable smart agriculture, fostering the exchange of traditional wisdom, modern technology, and social innovation. The meaningful participation of local communities ensures that agricultural technologies—particularly those involving beneficial microbes and sensor systems—are adapted to the social, cultural, and environmental contexts in which they are implemented. When communities are directly involved in the design, testing, and implementation of new agricultural tools, they develop a stronger sense of ownership, which in turn enhances adoption rates and long-term sustainability. Initiatives such as “Maize Demo” project exemplify how science can actively engage society beyond technical boundaries. This program encourages communities to reflect deeply on the role of science in daily life and its interaction with indigenous knowledge systems. Through interactive activities, such as the “Maize Along the Wall” events, participants share oral histories, music, and personal experiences that connect agricultural innovation with cultural identity. These engagements underscore the enduring value of indigenous agricultural practices—including seed selection, soil stewardship, and biodiversity conservation—demonstrate how they can be effectively integrated with emerging scientific tools such as IoT-based monitoring and microbial biofertilizers [10,11,23]. By blending storytelling with scientific awareness, such community-centered initiatives bridge the gap between traditional ecological knowledge and contemporary smart-farming technologies. They cultivate a shared understanding of the cultural and ethical responsibilities societies hold toward future generations, emphasizing that sustainable progress in agriculture must honor both scientific innovation and ancestral wisdom. Ultimately, participatory frameworks like these build public trust in science, strengthen local capacity for innovation, and ensure that smart agriculture evolves as an inclusive, community-driven movement [18,20].

### 11.2. Education and Training

**Figure 6:**
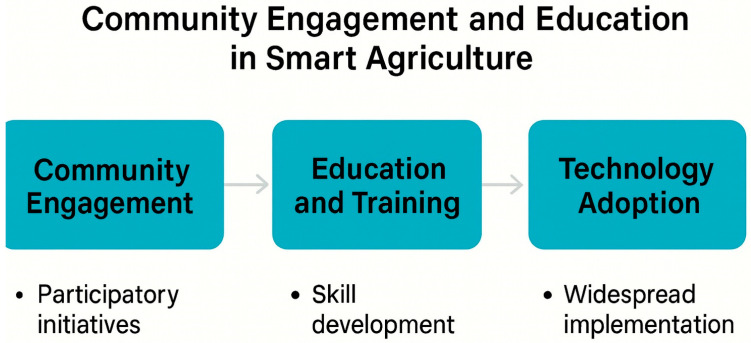
Conceptual framework illustrating Community Engagement and Education as integral components of Smart Agriculture. The diagram depicts three interconnected pillars—Community Engagement, Education and Training, and Smart Technology Adoption—linked through bidirectional arrows representing the continuous exchange of knowledge, experience, and innovation. The Community Engagement component emphasizes participatory approaches, integrating traditional agricultural knowledge, local customs, and stakeholder collaboration to strengthen ownership and trust. Education and Training focus on building technical capacity through farmer workshops, digital literacy programs, and field demonstrations, enabling the effective use of modern agro-technologies. The Smart Technology Adoption pillar encompasses the implementation of IoT-based sensors, data analytics platforms, and microbial innovations (e.g., AMF biofertilizers, Trichoderma-based biocontrols) to achieve data-driven and sustainable farming practices. Together, these pillars create a feedback-driven ecosystem that empowers farmers, enhances knowledge transfer, and accelerates inclusive agricultural transformation aligned with sustainability and resilience goals.

The successful implementation of smart agriculture fundamentally depends on a skilled and knowledgeable workforce capable of integrating advanced technologies with sustainable biological practices. Smart-farming systems—comprising interconnected sensors, data-driven analytics, microbial inoculants, and precision machinery—require specialized training to ensure their accurate, safe, and effective operation. Accordingly, education and capacity-building initiatives are essential to maximize the socioeconomic and environmental benefits of these innovations. A wide range of educational models and learning frameworks has emerged to meet the growing demand for smart-agriculture expertise. These include mobile- and e-learning programs, living laboratories, and hands-on training centers that promote continuous skill development. For instance, the Front Range Smart Agriculture Innovation Hub at Colorado State University offers practical learning opportunities through laboratory and field sessions focusing on farming equipment, drones, and proximal sensing technologies. Similarly, the Production Agriculture Risk Management Education Program, jointly organized by Purdue University and the University of Idaho, equips farm families with tools and strategies for informed decision-making, thereby reducing operational risks and enhancing farm resilience [55,72]. At the international level, projects such as SUNSpACe (Scalable User-centric Smart Farming Network for Smart, Precision and Automated Cultivations) are driving knowledge dissemination through mobile-learning platforms, localized workshops, and laboratory-based activities, particularly across developing regions. These programs aim to democratize access to smart-agriculture technologies by providing science-based training that is tailored to local contexts and responsive to farmer needs. Educational and extension systems play a critical role in facilitating the technology transfer of smart-farming innovations, ensuring that practitioners—from smallholders to industrial producers—can interpret sensor data, manage microbial applications, and optimize input use effectively. Universities and research institutions serve as pivotal intermediaries, delivering this knowledge through workshops, seminars, and extension programs that bridge research innovation with practical field application. Moreover, the integration of digital monitoring and control technologies has transformed agricultural extension services. Site-specific extension models, powered by remote sensing and ICT tools, now complement traditional advisory systems by offering real-time, tailored recommendations specific to farm location, crop types, and management practices [55,72]. This shift from generalized guidelines to data-driven, localized decision support ensures that smart agriculture not only boosts productivity but also promotes sustainability, knowledge equity, and long-term capacity development within the global agricultural community [11,22,48]. The interrelationship between community engagement, education, and smart-technology adoption is illustrated in Figure 6.

## 12. Technological Innovations in Microbial Applications

**Figure 7:**
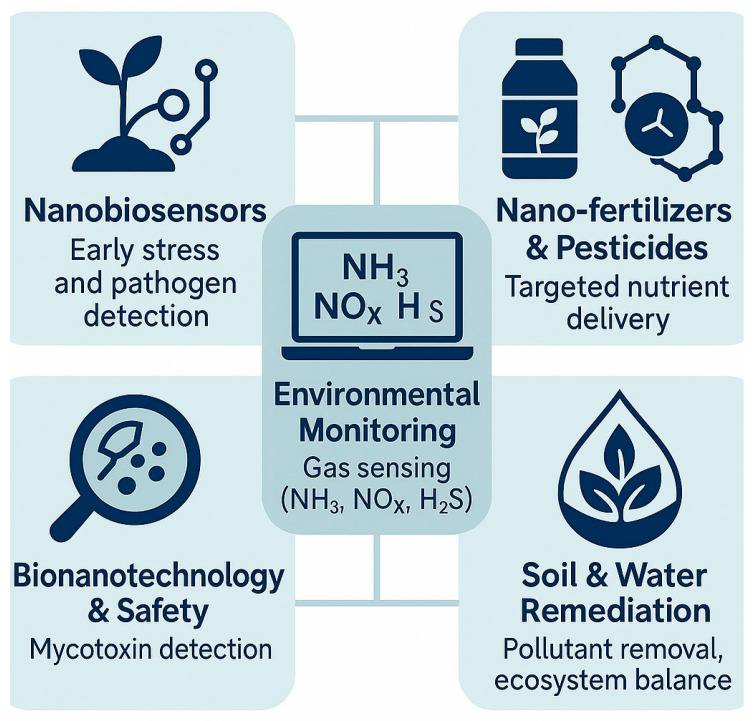
Conceptual framework illustrating the integration of biotechnology, nanotechnology, and smart sensing systems in sustainable smart agriculture. The diagram depicts the convergence of biological innovation (e.g., genetically enhanced microbes, RNA-based biocontrol agents, and molecular diagnostics), nanoscale engineering (e.g., nano-fertilizers, nano-pesticides, and biosensors), and advanced digital sensing (e.g., IoT-based environmental and physiological sensors). Arrows and connecting loops indicate multidirectional interactions among these domains, emphasizing real-time feedback, data integration, and adaptive management. This triadic integration enables precise nutrient delivery, early pathogen detection, reduced chemical inputs, and improved stress resilience. Together, these technologies form the foundation of next-generation agriculture that is efficient, sustainable, and environmentally intelligent, aligning biological and technological innovations for global food security.

Technological advancements in agriculture have rapidly accelerated the development of innovative food-production strategies, marking a paradigm shift toward biologically integrated and data-driven systems. These emerging approaches combine molecular biology, bioinformatics, and nanotechnology to enhance plant–microbe interactions, optimize microbial functionality, and improve the targeted delivery of beneficial microorganisms within agricultural environments. Collectively, these innovations form a cornerstone of next-generation smart agriculture, complementing sensor-based management systems and data-driven decision tools that drive sustainable intensification [10,11,18]. One major area of progress involves the genetic modification, transfer, and editing of genes that regulate plant–microbiome communication pathways. These molecular innovations improve microbial colonization, strengthen plant–microbe symbiosis, and enhance adaptive responses under variable environmental conditions [11,15]. Compared with traditional transformation or direct inoculation methods, engineered microbial symbioses demonstrate superior efficiency and broader functionality in promoting plant growth, nutrient acquisition, and yield stability [20]. Furthermore, the precise identification and characterization of key microbial taxa have enabled targeted applications based on specific crop species, soil types, and developmental stages, allowing for adaptive, precision-based agricultural management [10,22]. Recent breakthroughs in photosynthetic and metabolic engineering have expanded the frontiers of microbial biotechnology. The discovery and synthetic design of far-red-absorbing, light-harvesting pigments now allow crops to utilize previously untapped regions of the solar spectrum; thereby increasing photosynthetic efficiency and energy capture [23]. These discoveries pave the way for the development of artificial photosynthetic systems, in which phototrophic microorganisms—such as cyanobacteria or green algae—are strategically attached to plant surfaces or root zones without disrupting nutrient uptake. This configuration effectively enhances photosynthetic capacity and carbon assimilation, improving plant productivity under stress conditions [11,27]. Equally promising are innovations in artificial soil systems and bioengineered substrates, which foster microbial colonization, facilitate nodulation, and promote symbiotic nitrogen fixation. Such engineered microenvironments mimic the structure and function of natural soils while maximizing microbial processes, providing a stable and reproducible platform for sustainable crop production [10,18,20]. These systems are particularly valuable in degraded or saline soils, where microbial restoration of soil fertility is essential for climate-resilient farming [21]. Nanotechnology is also revolutionizing microbial formulation and delivery. Nanocarriers and nanoencapsulation systems improve the stability, bioavailability, and controlled release of beneficial microbes, extending their shelf life and ensuring efficient colonization of target plant tissues or rhizospheres [11,22,32,73]. Moreover, nanoparticle-based delivery platforms enhance communication between microbial inoculants and plant roots, improving uptake efficiency and facilitating rapid responses to biotic and abiotic stress. These nano-enabled formulations also exhibit high compatibility with sensor-integrated systems, enabling real-time monitoring of microbial activity and plant health under field conditions [22,27,32]. Alongside nanotechnology, artificial intelligence (AI) and bioinformatics-driven tools are becoming integral to microbial innovation in agriculture. Machine-learning algorithms can analyze complex datasets encompassing soil parameters, climatic variables, and microbiome profiles to identify optimal microbial inoculants and predict their field performance. A recent AI-assisted platform [56] demonstrated how predictive models can recommend crop-specific microbial consortia and application timings by integrating sensor-derived data such as soil moisture, temperature, and nutrient availability. These AI-enhanced frameworks enable adaptive management of microbial formulations, creating dynamic feedback loops that connect inoculant performance with real-time environmental data. By merging AI analytics with nanosensor feedback, agricultural systems are evolving toward intelligent, self-optimizing biofertilization and disease-suppression strategies. Overall, the smart integration of biotechnology, nanotechnology, and microbial ecology provides a comprehensive and scalable approach to precision microbial management. These innovations not only deepen our understanding of microbe–plant interactions but also enable reproducible, sustainable, and environmentally friendly agricultural solutions aligned with the principles of smart, data-driven, and climate-resilient farming [10,11,18,20]. The conceptual integration of these technological domains—biotechnology, nanotechnology, and smart sensing—is illustrated in Figure 7.

### 12.1. Biotechnology in Microbial Enhancement

Beneficial microbes play an indispensable role in sustainable agriculture, supporting plant growth and productivity even under environmentally adverse conditions. Recent advances in genetic engineering, systems biology, and synthetic biology have provided powerful tools for harnessing and enhancing microbial capabilities, enabling the optimization of crop performance and soil health [10,11]. Among the most extensively studied groups of beneficial microorganisms are plant growth-promoting rhizobacteria (PGPR), rhizobia, mycorrhizal fungi, and endophytic microbes. These organisms serve as biofertilizers, biopesticides, biocontrol agents, bioremediators, and bioenergy producers, collectively contributing to improved nutrient uptake, enhanced stress tolerance, and greater ecosystem stability [15,18,20]. A recent global market survey, encompassing over 40,000 plant growth-promoting microbial inoculant technologies, estimated an economic value of USD 1.76 billion, with annual growth rate exceeding 11.5% in 2019. This expansion reflects the rising global demand for eco-friendly microbial technologies, driven by increased public awareness of environmental sustainability and the need for alternatives to chemical inputs [15,18]. Despite over a century of microbial use in agriculture, the full technical potential of microbial tools has only recently been realized through the integration of analytical, genomic, and computational platforms. These platforms now allow for high-resolution characterization of microbial interactions, metabolic networks, and mechanistic pathways that drive plant–microbe symbioses [11,22]. Plant growth-promoting microbes encompass a diverse taxonomic spectrum, including PGPR, root-nodulating bacteria, mycorrhizal fungi, cyanobacteria, and actinomycetes, which collectively improve nutrient acquisition and confer resistance to both biotic and abiotic stresses [10,23]. Nevertheless, inconsistent efficacy under variable field conditions remains a major challenge to their widespread adoption. Such inconsistencies arise from complex biotic and abiotic interactions within diverse agroecosystems, highlighting the need for refined experimental frameworks that accurately link microbial performance with environmental parameters [15,20]. Several key scientific challenges persist in the field of microbial biotechnology, including: Deciphering plant–microbiome signaling networks, defining core and accessory microbiota, understanding microbe–microbe interactions dynamics, optimizing formulation processes and inoculation timing, and enhancing competitive colonization and persistence in soil environments [11,20,22]. Addressing these challenges demands coordinated communication among research institutions, governmental agencies, industries stakeholders, and funding bodies to sustain investment, innovation, and policy support for microbial biotechnology. From a commercial perspective, microbial products are best positioned as complementary technologies to traditional nutrient and pest-management programs, rather than as direct replacements for chemical inputs [17,18,74]. Their successes metrics extend beyond mere productivity gains to include reductions in agrochemical dependency, improvements in soil quality, and enhanced economic, environmental, and social outcomes—all of which align with the global goals of sustainable agriculture and the principles of smart-farming systems [5,11,18].

### 12.2. Nanotechnology Applications

Nanotechnology offers transformative possibilities for advancing smart and sustainable agriculture, reshaping how food systems manage inputs, monitor stress, and protect ecosystems. Its applications range from real-time sensing and targeted nutrient delivery to environmental remediation, positioning nanotechnology as a critical interface between microbial biotechnology and precision agriculture [10,11]. One of the most significant innovations in this field is the development of nanobiosensors capable of detecting early-stage plant stresses—including nutrient deficiencies, drought, or pathogen attacks—long before visible symptoms appear. These tools enable proactive management decisions, minimizing yield losses and improving crop resilience. By providing continuous, field-based diagnostics, nanobiosensors strengthen decision-support systems in precision farming, improving the timing and efficiency of microbial inoculant and fertilizer applications [23,75]. In parallel, nano-enabled fertilizers and pesticides-including nanoparticle-based formulations-facilitate site-specific and controlled delivery of essential nutrients and active ingredients. This innovation enhances nutrient-use efficiency, reduces application frequency, and substantially decreases chemical leaching and runoff. As a result, resource utilization becomes more efficient, while mitigating soil and water pollution associated with conventional agricultural practices [75,76]. Nanobiosensors, when embedded within smart-farming networks, can monitor a wide array of agro-environmental parameters, including nutrient concentrations, metabolite levels, pathogen presence, soil moisture, and temperature-transmitting real-time data to farmers for adaptive field management. For instance, electrochemically functionalized carbon nanotube and metal nanoparticle-based sensors can detect trace gases such as ammonia (NH_3_), nitrogen oxides (NO_X_), and hydrogen sulfide (H_2_S)—key indicators of soil and air quality—thereby assisting in pollution control and crop-health assessment [76]. Recent breakthroughs in bionanotechnology have accelerated the creation of high-sensitivity biosensors capable of detecting mycotoxins and other hazardous analytes that threaten crop safety and human health. Beyond monitoring applications, nanomaterials also offer promising solutions for soil and groundwater remediation. Nanoscale oxides and biogenic nanoparticles can immobilize or degrade pesticide residues, heavy metals, and excess nutrients, thereby contributing to the restoration of ecological balance [77]. Despite their immense potential, the successful deployment of nanotechnology in agriculture depends on farmer education, capacity-building and comprehensive field validation. Training programs are essential to familiarize growers with the operation, safety, and economic advantages of nanotechnology-based systems. Furthermore, extensive field trials are required to validate laboratory findings under real-world cultivation conditions, ensuring scalability, cost-effectiveness, and environmental safety [23,78]. Collaborative engagement among research institutions, industry stakeholders, and policymakers will be crucial to ensure that nanotechnology-driven agriculture evolves in a responsible, equitable, and sustainable manner—supporting the broader goals of climate-smart, eco-efficient, and data-enabled farming systems.

## 13. Regulatory Framework for Smart Agriculture

The deployment and commercialization of plant-beneficial microorganisms in agriculture remain constrained by regulatory frameworks that were originally designed for chemical pesticides rather than for biological control agents [59]. Within the European Union (EU), for example, the current pesticide registration system does not adequately account for the unique biological properties, mechanisms of action, or ecological behaviors of microbial control agents. Consequently, evaluation procedures often remain misaligned with microbial characteristics, resulting in unnecessary delays, increased costs, and the inefficient use of administrative resources [60]. These limitations underscore the urgent need to establish dedicated regulatory systems or specialized evaluation units equipped with expertise in plant-beneficial microorganisms. Such structures would enable risk-proportionate assessments, streamlined authorization processes, and more precise biosafety evaluations, thereby supporting innovation in microbial technologies while ensuring public safety and environmental protection. Potential improvements could be achieved either through incremental adaptations of current frameworks or, alternatively, through comprehensive legislative reforms specifically tailored to microbials agents [61]. Given the complexity and extended timelines associated with regulatory reform, short-term priorities should focus on updating risk-assessment protocols, harmonizing data requirements, and recognizing the inherently lower hazard potential of beneficial microbes compared to synthetic agrochemicals. In the long-term, effective strategies must aim to build institutional expertise, create supportive policy environments, and foster interagency collaboration among agricultural, environmental, and public health authorities [62]. Ultimately, the development of coherent and adaptive policies that facilitate the responsible advancement of microbial products—including biofertilizers, biopesticides, and biostimulants—is essential to achieving the overarching goals of smart, sustainable, and climate-resilient agriculture.

### 13.1. Current Regulations

Microbial technologies provide sustainable alternatives to chemical-based agricultural practices, while simultaneously enhancing plant growth, productivity, and resilience to stress [59]. However, regulatory frameworks have not evolved at the same pace as technological and industrial innovations, thereby restricting the introduction and commercialization of novel microbial solutions. Within the European Union (EU), microorganisms proposed for plant pest control are currently regulated under the Plant Protection Products (PPP) framework, which mandates rigorous pre-market approval. This process requires comprehensive and often costly risk assessments, along with compliance to strict data and testing requirements. While these evaluations are essential for ensuring biosafety, they can pose substantial challenges, particularly for small and medium-sized enterprises (SMEs) attempting to bring microbial products to market [79]. In response to the growing demand for microbial PPPs, the EU has recently introduced updated data requirements and evaluation principles designed to simplify authorization procedures while maintaining high safety standards. Notably, microbial PPPs that do not contain transferable antimicrobial resistance genes may now be classified as low-risk products, allowing for shorter and less expensive approval processes [79]. In contrast, plant biostimulants and microorganisms that primarily enhance abiotic stress tolerance currently lack a harmonized regulatory framework at the EU level. Their regulation varies widely among member states and typically falls under national fertilizer legislation, which differs significantly in definitions, safety evaluation criteria, and authorization processes [80]. This fragmented regulatory landscape leads to inconsistencies in market access and limits the scalability of innovative microbial technologies across the EU. To address these issues, several European and international initiatives are now focused on the harmonization of microbial regulations. These efforts seek to standardize definitions, streamline approval pathways, and enhance policy coherence between microbial PPPs, biofertilizers, and biostimulants. Achieving this regulatory alignment will be crucial for stimulating innovation, ensuring biosafety, and advancing smart, sustainable, and climate-resilient agricultural systems [81].

### 13.2. Future Policy Directions

**Figure 8:**
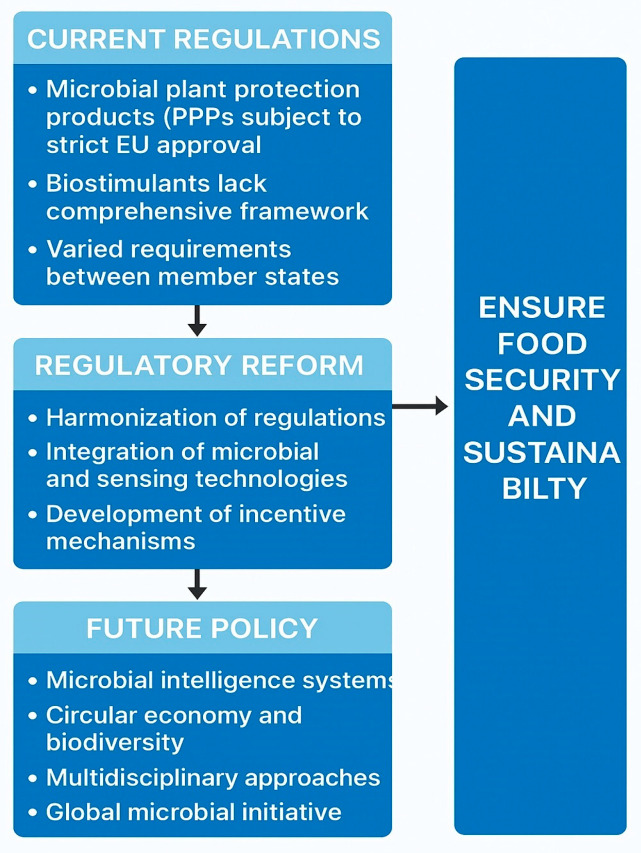
Regulatory and policy roadmap for smart agriculture, illustrating the progressive transition from current European Union (EU) frameworks toward future global strategies that integrate microbial technologies, sensor systems, and sustainability-oriented policies. The figure outlines three developmental stages—Current Regulations, Regulatory Reform, and Future Policy Directions—each connected by directional arrows representing policy evolution and feedback mechanisms. The Current Regulations stage highlights existing EU directives governing agrochemical use, biosafety, and environmental protection. The Regulatory Reform phase emphasizes harmonization of microbial inoculant standards, the inclusion of sensor-based monitoring in compliance systems, and the incorporation of digital traceability tools. The Future Policy stage projects a globally coordinated framework aligning biotechnology innovation, nanotechnology applications, and smart sensing networks with the United Nations Sustainable Development Goals (SDGs). This roadmap underscores how integrative and adaptive policy design can foster innovation, ensure biosafety, and advance sustainability in the era of smart agriculture.

Ensuring the global supply of secure, safe, and nutritious food is among the most urgent challenges of the 21st century. Meeting this demand requires innovative production systems that balance sustainable resource use, minimize waste, and reduce carbon emissions, while maintaining high levels of productivity and quality. An effective policy framework must recognize the complementary roles of beneficial microbes and sensor technologies in achieving these objectives. Microbes enhance nutrient acquisition, promote plant growth, and strengthen resistance against pests and diseases, whereas sensor-based monitoring systems provide real-time data of soil, water, and crop parameters. When integrated, these technologies enable the production of nutritionally enriched crops with higher yields and lower input dependency, thereby advancing the principles of sustainable intensification [82]. Sustainability in agriculture depends not only on maintaining productivity but also on preserving biodiversity and ensuring the adaptive capacity of agroecosystems under changing climatic conditions. Thus, future agricultural policies should aim to integrate microbial biotechnology and geospatial sensing within a comprehensive governance framework that promotes the circular economy, biodiversity conservation, and the valuation of ecosystem services and rural environmental goods [82]. Progress toward these goals will be propelled by the development of advanced microbial formulations and next-generation biosensors capable of monitoring microbial activity, soil nutrient fluxes, and ecosystem dynamics with unprecedented precision. Simultaneously, incentive-based policy mechanisms will be necessary to facilitate widespread adoption among farmers and industry stakeholders. Such mechanisms might include subsidies for microbial biosensor systems, recognition of low-carbon farming practices, and targeted funding for microbial R&D initiatives. Achieving an effective microbial intelligence system for sustainable smart agriculture will require the convergence of multiple scientific disciplines—including biology, chemistry, materials science, and process engineering—to co-design microbially based smart formulations, sensor networks, and data-driven analytical workflows. Beyond agriculture, these integrated systems can also be applied across industrial and environmental sectors to improve the sustainable delivery of macro- and micronutrients at multiple spatial and production scales. Importantly, future policy frameworks must position microbiology as a central pillar within the STEM disciplines, reflecting its crucial role in environmental stewardship, sustainable production, and global food security. Establishing a global microbial initiative for smart agriculture would represent a transformative step toward achieving long-term food security and ecological resilience [11,18]. Ultimately, sustained food production across diverse agroecological zones will depend on advanced irrigation systems, microbial inoculation technologies, and the deployment of plant- and soil-beneficial microbes that improve crop adaptability to emerging environmental stresses. The integration of these interrelated technologies provides a viable pathway toward sustainable global food production, ensuring both ecological balance and human well-being [18] (Figure 8).

## 14. Conclusions

Modern agriculture faces unprecedented and complex challenges that are expected to persist throughout the twenty-first century. Overcoming these challenges requires not only advances in scientific research but also the development of a comprehensive framework for the implementation of sustainable smart agriculture. The integration of beneficial microbes and sensor technologies provides a transformative pathway toward achieving this vision by fostering productivity, resource efficiency, and ecological stability within contemporary agroecosystems. Beneficial microbes and microbial inoculants, known for their multifunctional plant growth-promoting properties, are increasingly recognized as indispensable tools for enhancing global agricultural productivity. Beyond improving crop yields, these microorganisms deliver critical ecosystem services such as soil fertility restoration, bioremediation of contaminants, nutrient cycling, and enhanced stress tolerance. In parallel, sensor technologies have revolutionized data-driven agriculture by enabling real-time monitoring of key environmental parameters, including soil moisture, nutrient availability, temperature, and pest dynamics. These data streams empower farmers to make precise, evidence-based management decisions, optimizing resource use while minimizing environmental impacts. The future of sustainable agriculture lies in the synergistic fusion of microbial ecology and precision sensing, leading to integrated platforms that combine microbial community data with sensor-based analytics. Such systems are capable of generating dynamic environmental maps and feedback-controlled management strategies, thereby facilitating both short-term operational efficiency and long-term ecological resilience. However, realizing this potential requires overcoming key barriers related to cost, scalability, interoperability, and regulatory harmonization. The success of smart agriculture ultimately depends on collaborative engagement among scientists, policymakers, farmers, and consumers, who must collectively adopt an integrated approach that unites biological sciences, digital technologies, and policy innovation. By fostering interdisciplinary collaboration, the agricultural sector can transition toward a climate-resilient, resource-efficient, and socially equitable model of food production—ensuring global food security and environmental stewardship for generations to come [11,18]. To guide future progress and practical implementation, the following key actions are recommended: (i) establish interoperability standards to enable seamless data integration across microbial, sensor, and decision-support platforms; (ii) develop validated microbial–sensor performance metrics to benchmark biological efficacy, soil health improvement, and yield outcomes under field conditions; (iii) promote coordinated regulatory reform to streamline approval processes for bioinoculants and smart-farming technologies, ensuring efficiency and biosafety; (iv) strengthen farmer education and training programs that integrate microbial technologies with digital and precision-farming tools; and (v) foster cross-disciplinary and international collaboration among researchers, industries, and policymakers to accelerate innovation, enhance global scalability, and drive sustainable transformation.

## Figures and Tables

**Table 1:** Major groups of beneficial microbes and their primary mechanisms of action in sustainable agriculture.

Microbial Group	Representative Genera/Species	Primary Mechanism(s) of Action	Agronomic Impact	Selected References
Plant Growth-Promoting Rhizobacteria (PGPR)	*Bacillus*, *Pseudomonas*, *Azospirillum*, *Burkholderia*	Nitrogen fixation, phosphate solubilization, siderophore and phytohormone production (auxins, gibberellins, cytokinins), induction of systemic resistance	Improved nutrient uptake, enhanced root development, increased biomass and yield	[6,10,11,15,16]
Rhizobia	*Rhizobium*, *Bradyrhizobium*, *Sinorhizobium*	Symbiotic nitrogen fixation in legumes; modulation of nodulation signaling	Increased nitrogen supply and legume productivity; reduced need for chemical N fertilizers	[10,15,16]
Arbuscular Mycorrhizal Fungi (AMF)	*Glomus*, *Gigaspora*, *Rhizophagus*, *Funneliformis*	Enhancement of P, Zn, and water uptake; improvement of soil aggregation; stress alleviation	Greater nutrient efficiency, drought/salinity tolerance, and soil health	[7,10,11,18]
*Trichoderma* spp.	*Trichoderma harzianum*, *T. atroviride*, *T. asperellum*	Antagonism via mycoparasitism, antibiosis, hydrolytic enzyme production, VOC emission	Reduced disease incidence, improved root vigor, induced systemic resistance	[6,11,18]
Phosphate-Solubilizing Bacteria (PSB)	*Bacillus*, *Pseudomonas*, *Penicillium*, *Aspergillus*	Secretion of organic acids and phosphatases to mobilize insoluble P	Improved phosphorus bioavailability and fertilizer-use efficiency	[14,15,16]
Endophytic Microbes	*Serendipita indica*, *Piriformospora*, *Clonostachys*, *Burkholderia* spp.	Production of growth regulators, stress-protection metabolites, and antagonistic enzymes within host tissues	Enhanced plant tolerance to abiotic stress, improved growth and defense	[10,11,18]

**Table 2:** Representative global case studies (field and pilot validations) illustrating how microbial innovations and sensor technologies jointly enhance sustainable agricultural practices.

Crop/System	Region/Country	Microbial Technology	Sensor/Monitoring Tool	Key Outcome	Validation Type	Reference
Banana	Asia, East Africa	Microbial consortia + biocontrol	IoT field sensors	Reducing Disease, Increasing soil microbiota	Pilot field trials	[6]
Soybean	Brazil	*Bradyrhizobium*, *Azospirillum*	Soil nutrient sensors	Reducing chemical N by 30%	Commercial application	[41]
Vineyard/Wheat	Europe	*Gigaspora rosea* + *Penicillium bilaiae*	Nutrient sensors	Increasing P uptake, Increasing growth	Greenhouse + multi-site field	[41]
Fruit Orchards	USA	Biopesticide + microbial control	Semios IoT network	Reducing pesticide use 25%	Commercial deployment	[40]

**Table 3:** Key challenges and potential mitigation strategies in the implementation of smart agriculture.

Challenge Category	Specific Issues	Impact on Smart Agriculture	Possible Mitigation Strategies	Reference
Biological	Variable microbial performance in field conditions; interaction with native microbiota	Inconsistent inoculant efficacy and crop response	Use locally adapted strains; develop microbial consortia and stable formulations	[10,50]
Technical	Short shelf life, storage and transport limitations; sensor calibration issues	Reduced product reliability and monitoring accuracy	Encapsulation, freeze-drying, or carrier optimization; standardized sensor maintenance protocols	[11,53,55]
Economic	High cost of equipment and inoculants	Low adoption rate among smallholders	Subsidies, cooperative equipment sharing, development of low-cost IoT platforms	[52,54]
Regulatory/Institutional	Lack of product certification and extension support	Poor market confidence; limited farmer guidance	Establish regulatory frameworks; promote certification and training programs	[52]
Educational/Social	Limited farmer knowledge of microbial and sensor benefits	Reluctance to adopt new technologies	Demonstration plots, participatory workshops, and digital outreach tools	[11,52,54]

**Table 4:** Global and local case studies showcasing successful integration of microbial and sensor technologies for sustainable agriculture.

Region/Country	Technology/Approach	Key Microbes/Sensors Used	Outcome	Reference
Brazil	Biological nitrogen fixation in soybean	*Bradyrhizobium japonicum*	Reduced fertilizer input, higher yield	[41]
Asia (Japan/China)	Disease resistance in rice	*Sphingomonas melonis*	Enhanced resistance, reduced chemical fungicides	[4]
EU/USA	Phosphorus solubilization via microbial biofertilizer	*Penicillium bilaiae* + soil sensors	Improved P efficiency and soil fertility	[40]
Pakistan	Smart irrigation and energy-efficient farming for saffron	IoT sensors + beneficial microbes	Increased water- and nutrient-use efficiency, improved livelihoods	[11]
